# End of life care for people with severe mental illness: Mixed methods systematic review and thematic synthesis (the MENLOC study)

**DOI:** 10.1177/02692163211037480

**Published:** 2021-09-03

**Authors:** Deborah Edwards, Sally Anstey, Michael Coffey, Paul Gill, Mala Mann, Alan Meudell, Ben Hannigan

**Affiliations:** 1 School of Healthcare Sciences, College of Biomedical and Life Sciences, Cardiff University, Cardiff, UK; 2 Department of Public Health, Policy and Social Sciences, College of Human and Health Sciences, Swansea University, Swansea, UK; 3 Specialist Unit for Review Evidence, University Library Services, Cardiff University, Cardiff, UK; 4 Independent Service User Researcher, Caerphilly, UK

**Keywords:** Diseases, health services administration, intersectoral collaboration, mental disorders, neoplasms, systematic review, thematic synthesis

## Abstract

**Background:**

Parity of esteem means that end-of-life care for people with severe mental illness should be of equal quality to that experienced by all.

**Aim:**

To synthesise international, English language, research and UK policy and guidance relating to the organisation, provision, and receipt of end-of-life care for people with severe mental illness.

**Design:**

A mixed methods systematic review was conducted following the Evidence for Policy and Practice Information and Co-ordinating Centre approach and informed by a stakeholder group. We employed thematic synthesis to bring together data from both qualitative and quantitative studies, and from non-research material. We assessed the strength of synthesised findings using the Confidence in the Evidence from Reviews of Qualitative Research (CERQual) and Grading of Recommendations, Assessment, Development and Evaluation (GRADE) approaches.

**Data sources:**

Ten electronic databases were searched from inception to December 2019, along with 62 organisational websites. Quality appraisal was conducted using Critical Appraisal Skills Programme checklists or other study design-specific alternatives as necessary.

**Results:**

Of the 11,904 citations retrieved, 34 research publications were included plus 28 non-research items. The majority of research was of high or acceptable quality. An overarching synthesis including 52 summary statements, with assessments of confidence in the underpinning evidence, was produced using four themes: *Structure of the system; Professional issues; Contexts of care*; and *Living with severe mental illness*.

**Conclusions:**

Implications for services and practice reflect evidence in which there is a high degree of confidence. Partnership should be developed across the mental health and end-of-life systems, and ways found to support people to die where they choose. Staff caring for people with severe mental illness at the end-of-life need education, support and supervision. End-of-life care for people with severe mental illness requires a team approach, including advocacy. Proactive physical health care for people with severe mental illness is needed to tackle problems of delayed diagnosis.

**What is already known about the topic?**

- In high, middle and low-income countries people with mental illness have both poorer physical health and reduced life expectancy compared to the general population.
- Premature death in people with severe mental illness has been described as a human rights disgrace, reflected in renewed efforts to promote standards of care for people with severe mental illness which are as good as they are for people with physical health problems.
- In this context, evidence is needed to underpin improvements in end-of-life care for people with severe mental illness.

**What this paper adds?**

- This paper reports on a mixed methods systematic review and thematic synthesis of international English language research, plus UK policy and guidance, on end-of-life care for people with severe mental illness.
- The structure of mental health and end-of-life care systems means that people with severe mental illness at the end-of-life often have difficulty getting the services they need, whilst the education and practice of professionals in each of these two systems can create barriers to the provision of care.
- People with severe mental illness at the end-of-life are a particularly vulnerable group, having complex needs which are both difficult to assess and meet.

**Implications for practice, theory or policy?**

- Close partnerships are needed between the mental health and end-of-life care systems, enabling people with severe mental illness to die where they choose.
- Health and social care staff looking after people with severe mental illness at the end-of-life need education, support and supervision.
- Services for people with severe mental illness at the end-of-life require a team approach including advocacy, and proactive physical health care is needed for people with severe mental illness to tackle problems associated with delayed diagnosis.

## Introduction

In all parts of the world it is known that people with mental illness have poorer physical health and reduced life expectancy compared with the general population.^1^ Premature death in this group has been described as a human rights disgrace,^2^ driving international and national efforts to tackle disparities. The phrase ‘parity of esteem’, used across the world^3^ and first introduced in the UK in England’s Health and Social Care Act 2012,^4^ refers to the principle that people with mental health difficulties should have access to care of the same standard and timeliness as that enjoyed by people with physical health difficulties. International guidance^5^ emphasises the importance of high-quality palliation and support for all, irrespective of underlying condition. By extension this includes the expectation that care for people with pre-existing severe mental illness who go on to develop end-of-life conditions (such as incurable cancer and/or end-stage organ disease) should be as good as it is for everyone else.

Longstanding concerns remain, however, that the end-of-life needs of people with severe mental illness are acknowledged either poorly or not at all, leading to the prospect of ‘disadvantaged dying’.^6,7^ Initial research observations informing the project reported on here suggest inadequacies in care and disparities associated with people’s experiences of mental illness,^8–13^ supporting the case for a high-quality evidence synthesis beginning a programme of work to improve and evaluate care. Whilst a number of earlier reviews in this area have been conducted these are variously in need of updating,^12,14^ have addressed a relatively narrow range of issues such as health care access and place of death,^15^ or the use of palliative care tools and interventions,^16^ are non-systematic narrative reviews^17–20^ or have been limited to only scoping what is known.^11,13^ The point has also been made that the voice of people with severe mental illness and their carers has largely been missing from existing work in this area.^11^

Preliminary scoping of the field therefore confirmed the timeliness and feasibility of a new, rigorous, evidence synthesis, and particularly an EPPI-Centre style review which is sensitive to the needs of stakeholders and which includes grey and non-research materials.^21^ In the larger project from which this paper is derived a mixed methods systematic review and thematic synthesis was therefore completed to address the following research question: ‘What evidence is there relating to the organisation, provision and receipt of care for people with severe mental illness who have an additional diagnosis of advanced, incurable, cancer and/or end-stage lung, heart, renal or liver failure and who are likely to die within the next 12 months?’

## Methods

The protocol is registered in PROSPERO (CRD42018108988), and reporting is in accordance with the PRISMA statement.^22^ The review followed Centre for Reviews and Dissemination^23^ guidance and incorporated stakeholder views following EPPI-Centre methodology.^21^

*Inclusion criteria*: We used the PICOS/PiCo framework to guide the inclusion criteria on population (P), intervention/phenomena of Interest (I), comparators (C), outcome (O), study design (S) and context (Co).

*Population*: Adults with severe mental illness who have an additional diagnosis of advanced, incurable, cancer and/or end-stage lung, heart, renal or liver failure and who are likely to die within the next 12 months. ‘Severe mental illness’, and related terms such as ‘serious’, or ‘serious and persistent’ mental illness, have been widely used around the world for years,^24^ and although lacking consensus definition broadly refer to people with experience of using specialist, secondary, community and/or hospital mental health services. Severe mental illness included, but was not limited to, people with diagnoses of schizophrenia, schizophrenia spectrum and other psychotic disorders, schizotypal and delusional disorders, bipolar affective disorder, bipolar and related disorders, major depressive disorder and disorders of adult personality and behaviour. End-of-life care is more clearly defined, referring to the care of people who are likely to die within the next 12 months.^25^

*Intervention/Phenomena of interest*: Service organisation, provision and receipt of end-of-life care along with the views and experiences of service users, families and health and social care staff.

*Comparators*: None.

*Outcomes*: Services, processes and interventions facilitating and hindering the provision of high-quality, accessible, equitable and acceptable end-of-life care to people with severe mental illness along with the views and experiences of service users, families and health and social care staff.

*Study design*: Quantitative and qualitative research, and non-research material (UK-only policies and guidelines, reports of practice initiatives and clinical case studies). UK-only policies and guidance were sought reflecting the project’s UK funding, and a recognition that many decision-makers reading the larger report from which this key findings paper is derived will be UK-based. Findings from our synthesis of clinical case studies will be presented in a further publication.

*Context*: End-of-life care provided in hospitals, hospices and other institutional settings (such as care homes, prisons and hostels) and care provided in the home and via outreach to people who may also be homeless. Research studies from the Organization for Economic Co-operation and Development countries as these were deemed to be socially and economically comparable to the UK.

### Exclusion criteria

Material addressing the following was excluded:

- Mental health problems subsequent to terminal illness;
- End-of-life care for people whose mental health problems reflected substance use other than where these coexisted with severe mental illness;
- End-of-life care for people with dementia or other neurodegenerative diseases except where these coexisted with severe mental illness.

### Stakeholder engagement

Project team members worked with an independently chaired stakeholder advisory group (SAG). This group included policy advisors, senior practitioners, and researchers from the end-of-life care and mental health practice fields, along with public and patient representatives. Examples of SAG involvement in decision-making are given below.

### Searching for relevant material

Reflecting imprecisions in the use of the phrase ‘severe mental illness’, and the need to also specify parameters for searches in the end-of-life field, project and SAG members in their first combined meeting discussed candidate keywords and search strategies and refined the boundaries of the searches to be adopted. In the case of the mental health-related arm, a series of terms were purposefully included which reflected diagnoses typically associated with psychosis (e.g. schizophrenia and bipolar disorder), recognising that diagnosis is often used as a shorthand to identify people with ‘severe mental illness’. Preliminary database searches designed to improve sensitivity and specificity informed a systematic search across 10 databases from inception to December 2019: MEDLINE; PsycINFO; EMBASE; HMIC, AMED; CINAHL; CENTRAL; ASSIA; DARE; and Web of Science. The search strategy was developed in Ovid Medline by an information specialist using a combination of text words and Medical Subject Headings (see Supplemental File 1), before adapting for use in the remaining databases. Searches were limited to English language publications.

Additional searches were: 62 targeted websites identified with the help of the SAG (e.g. belonging to mental health and end-of-life charities); Google;^26^ the contents pages for the last 2 years of identified journals; reference lists of included studies; and forward citation tracking. All retrieved citations were entered into EndNote™ [https://endnote.com/], and duplicates and references that did not meet the study’s inclusion criteria were removed.

### Screening

Citations were imported into Covidence™ [https://www.covidence.org/] and titles and abstracts assessed. Full texts which looked to meet inclusion criteria, or about which decisions could not be made due to insufficient information, were further screened by two reviewers using a standardised form with disagreements resolved with a third.

### Quality appraisal

Included studies were appraised by two reviewers (with disagreements resolved through involvement of a third) using the following:

- Qualitative studies: CASP checklists^27^
- Cross sectional designs: SURE checklist^28^
- Retrospective cohort studies: SIGN Checklist 3; Cohort Studies^29^

Policy and guidance documents were not appraised.

### Data extraction

Information (author, publication year, country, aim, setting, design, participant characteristics, outcomes, thematic findings) from included studies was extracted into tables^23^ and checked by a second reviewer. Where multiple publications from the same study were identified, data were reported as a single study. Summary statistics were extracted as reported across the original studies and included odds ratio, risk ratios and hazard ratios.

### Data analysis and synthesis

The search did not identify any intervention studies that met the inclusion criteria and as a result meta-analysis was not possible. We therefore employed thematic synthesis to bring together data from across both qualitative and quantitative studies and non-research material.^30^

The full text of all quantitative and qualitative research studies along with relevant extracts (addressing mental health and end-of-life care) from included policies and guidance were uploaded into NVIVO-12™ [https://www.qsrinternational.com/nvivo-qualitative-data-analysis-software/home]. Inductive data-driven codes were generated and attached to segments of material through the line-by-line reading of documents using NVIVO by two researchers. Codes were then grouped into meaningful candidate themes and sub-themes reflecting the overarching objectives of the project (and then finalised) with the wider team and the SAG.

### Assessing confidence

Confidence in the quality of synthesised evidence was assessed using either the GRADE^31^ approach (in the case of findings from cohort studies) or the CERQual^32^ approach (in the case of qualitative and non-intervention findings). GRADE involves judging the quality of a body of evidence using a four-point scale (‘high’, ‘moderate’, ‘low’ or ‘very low’) using these criteria: underpinning study design; risk of bias; impression; inconsistency; indirectness; and magnitude of effect. CERQual involves making judgements using the same four-point scale, and assessing the underpinning research using these criteria: coherence; methodological limitations; relevance; and adequacy.

## Results

**Figure 1.**
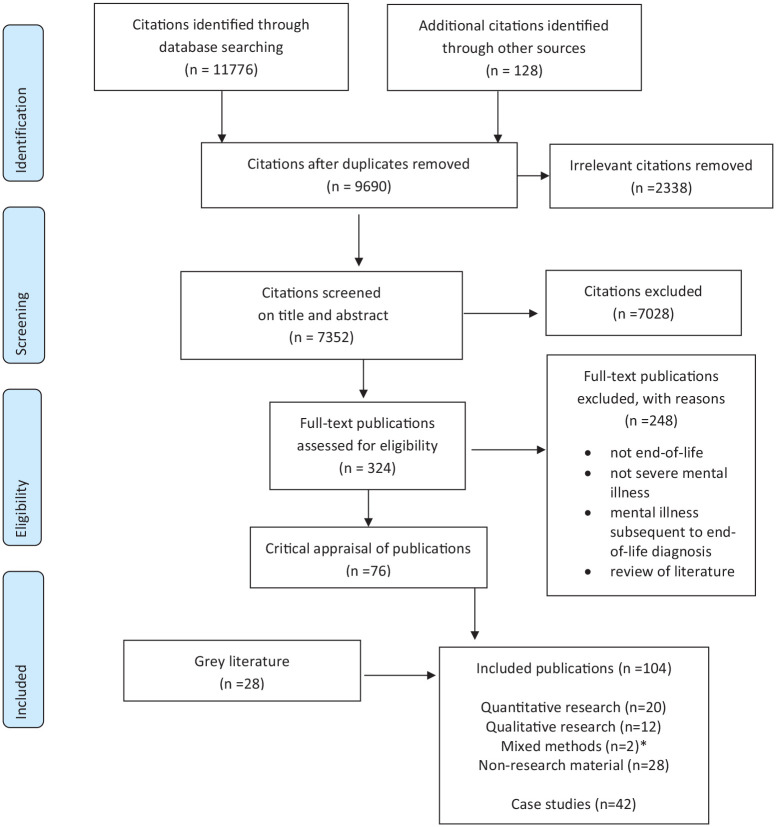
PRISMA flow chart. *The two mixed methods studies contributed both qualitative and quantitative data to the review.

The flow of citations through the overall review is shown in the PRISMA chart (Figure 1). Thirty-four publications reporting on 30 research studies and 28 pieces of non-research material were included. Of the research studies there were 19 quantitative studies (reported in 20 publications), nine qualitative studies (reported in 11 publications) and two mixed methods studies (for included studies tables, see Supplemental File 2).

### Description of research studies

The quantitative studies included retrospective cohort studies (*n* = 12, across 13 publications)^10,33–44^ and descriptive surveys (*n* = 7, across 8 publications).^45–52^ The qualitative studies included those using a non-specific qualitative descriptive approach (*n* = 5 across 7 publications),^53–59^ grounded theory (*n* = 2),^60,61^ ethnography (n = 1)^62^ and phenomenology (*n* = 1).^63^ One mixed methods study combined a medical records review, an educational evaluation with surveys and interviews, and the other combined a survey and interviews.^64,65^

Findings were reported across 10 countries, with studies from the US the largest group (*n* = 12, across 13 publications).^34,36,37,39,40,42,45–50,63^ Other countries included Canada (*n* = 4, across 5 publications),^10,41,43,51,62^ New Zealand (*n* = 1),^33^ Taiwan (*n* = 1),^38^ Australia (*n* = 4, across 6 publications),^44,53–57^ France (*n* = 1),^35^ Belgium (*n* = 1),^60^ The Netherlands (*n* = 2),^52,60,64^ UK (*n* = 3)^58,59,61^ and Republic of Ireland (*n* = 1).^65^

Publications appeared between 2003 and 2019, with data generated in a variety of settings including specialist palliative care, US Veterans Administration mental health services along with care homes, hostels and in the community. In 11 studies (across 12 publications) data were derived from the records of deceased people with diagnoses of severe and persistent mental illness,^33^ schizoaffective disorder,^10,35,36,38,41,44^ a pre-existing psychiatric illness,^40^ a mental health diagnosis,^37^ pre-cancer depression^42^ or post-traumatic stress disorder (PTSD).^39^ Seven studies (across 8 publications) directly involving patients with severe mental illness^34,43,47,48,51,59,60,62^ and a further 12 studies (across 14 publications) involved health care professionals who worked with patients with severe mental illness at the end-of-life.^46,49,50,52–58,61,63–65^

### Quality appraisal

Tables of the quality appraisal scores are presented in Supplemental File 3.

The quality of all included cohort studies was acceptable, indicating some flaws in the study design were present with an associated risk of bias. Seven of the cohort studies did not provide confidence intervals as part of the statistical analysis.^33,36,37,39,40,43,44^ For the descriptive surveys (*n* = 7) and the survey component of the mixed methods studies (*n* = 2), the majority (*n* = 7) were rated as high quality, meeting either 11 or all 12 of the checklist criteria. For the qualitative studies (*n* = 11) and the qualitative component of the mixed methods studies (*n* = 2) the quality of all except one was high, meeting at least nine of the 10 quality criteria.

### Thematic synthesis

**Figure 2.**
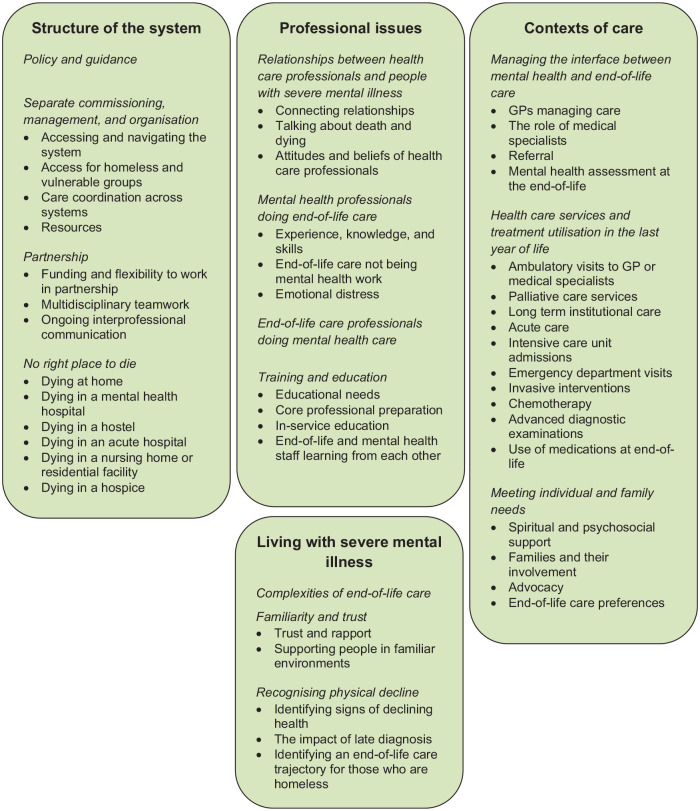
Summary of synthesis of research, policy and guidance.

The findings from the quantitative and qualitative research, and from the included policy and guidance materials, were synthesised and four themes created (see Table 1 and Figure 2).

**Table 1.** Themes and types of data included.

Theme	Type of data contributing to the theme
*Structure of the system* Addressing the broad shape and structure of the end-of-life and mental health care systems	Policy and guidance (*n* = 26)^66–91^ Cohort studies (*n* = 6)^35,36,40–43^ Descriptive studies (*n* = 3)^47,49,50^ Mixed methods studies (*n* = 1)^65^ Qualitative studies (*n* = 7)^54,56–59,62,63^
*Professional issues* Addressing practitioner-level issues	Policy and guidance (*n* = 10)^67,68,74,77–79,81,86,91,92^ Descriptive studies (*n* = 4)^49,50,52,65^ Mixed methods studies (*n* = 2)^64,65^ Qualitative studies (*n* = 11)^52–59,61–63^
*Contexts of care* Addressing the organisation, provision and receipt of care	Policy and guidance (*n* = 11)^25,67,68,73,74,77–79,81,85,91^ Cohort studies (*n* = 12)^10,33–35,37–44^ Descriptive studies (*n* = 5)^45–48,51^ Mixed methods studies (*n* = 1)^64^ Qualitative studies (*n* = 8)^56–63^
*Living with severe mental illness* Addressing the individual and social characteristics of people with severe mental illness	Policy and guidance (*n* = 8)^67–70,73–75,85^ Descriptive studies (*n* = 1)^47^ Mixed methods studies (*n* = 2)^64,65^ Qualitative studies (*n* = 8)^53,54,57–59,61–63^

An overarching summary and a set of 52 synthesis summary statements derived from both the descriptive quantitative and qualitative research, and from included policy and guidance documents, was produced with levels of confidence using the CERQual approach (see Supplemental File 4). Ninety-six summary statements organised by theme, and derived from the retrospective cohort research, were produced with levels of confidence using the GRADE approach (see Supplemental File 5). As the design of all the cohort studies was retrospective the ratings for evidence from each outcome generated using material from these were downgraded from ‘low quality’ to ‘very low quality’.^93^

#### Structure of the system

There is limited UK guidance relating to end-of-life and mental health care, and palliative care nurses report concerns over their legislative responsibilities (CERQual–very low).^58^ People with severe mental illness at the end-of-life who are additionally homeless, or from other particularly vulnerable groups, are often isolated from informal carers (ungraded–non research).^67,74,82^ They are also sometimes excluded from hospices and care homes, and professionals report difficulties in finding placements in these circumstances (CERQual–moderate).^59,62^ They can also become relatively invisible because of fragmented care systems and often have to act as their own care coordinators (CERQual–moderate).^58,59,62^

Practitioners recognise that the separate commissioning, management and organisation of mental health and end-of-life services means people with severe mental illness often have difficulty accessing and navigating the system (ungraded–non research),^69,78,88^ with multiple obstacles existing (CERQual–moderate).^53–55,57,65^ Coordinating high-quality integrated care, although difficult to achieve, is important (ungraded–non research).^67,78,84,85^ This is challenged, however, by the separation of services and professionals (CERQual–very low)^58^ and by the limited availability of services and trained practitioners able to meet need (CERQual–moderate).^46,49,64^ Ideas on improving the coordination of care often involve identifying staff with clear responsibilities (CERQual–moderate).^46,63,65^

Working in partnership across service boundaries is important, along with flexibility to facilitate end-of-life care (CERQual–very low).^46^ Poor working relationships and partnerships, influenced by reductions in funding and competitive commissioning, inhibit high-quality care (CERQual–moderate).^57,58,65^ Interagency and interprofessional working is important (ungraded-non research)^67,77,80,83,85,86^ along with ongoing communication (ungraded–non research)^67,78,80^ and professionals describe how both formal and informal multidisciplinary teamwork can improve outcomes (CERQual–moderate).^46,53–57^ Building partnerships and creating formal opportunities to meet and discuss care are essential and should include making information available to colleagues in primary care and social services (CERQual–high).^46,57,58,65^

People with severe mental illness at the end-of-life are able to stay at home, with support (ungraded–non research),^69^ and staff recognise that enabling people to die where they choose (often a home or home-like environment) is important, although staff also talk about how appropriate care is often lacking in all settings (CERQual–high).^57,63,65^ Findings are mixed regarding how likely people with severe mental illness are to have died at home (Grade–very low),^36,40,41^ but a consistent finding is that they are significantly more likely to have died in a nursing home or residential facility (Grade–very low).^36,40,41^ People with severe mental illness at the end-of-life are often transferred between services, and mental health staff rarely care for them as they are poorly equipped to meet their needs (CERQual–high).^58,63,64^

Multiple challenges to end-of-life care in hostels or supported accommodation exist, which include limited preparedness of staff, chaotic environments and concerns over both risks and medication storage (CERQual–moderate).^43,59^ Providing end-of-life care in the community, within mental health settings or homeless shelters, can be difficult and people with severe mental illness are frequently moved to acute settings which also brings challenges (CERQual–moderate).^56–58,62–65^ Hospice staff describe their workplaces as poorly prepared to care for homeless people at the end-of-life, and they require help to meet the needs of people with additional substance misuse and other problems (CERQual–moderate).^59,62^ No significant differences exist in the likelihood of people with or without severe mental illness dying in a hospice (Grade–very low), and findings are mixed regarding the likelihood of people with severe mental illness dying in hospital (Grade–very low).^40^

#### Professional issues

Forming relationships are important, although some mental health staff choose not to form close relationships with people with severe mental illness as they find it too upsetting when patients are moved for end-of-life care (CERQual–high).^53–55,57,63–65^ Supporting them to make decisions, including talking about death and dying, is recognised as important and requires good communication (ungraded–non research).^78,91^ Whilst some mental health staff find it difficult to talk about death and dying, those who do report that patients are receptive (CERQual–high).^52,57,58,64^ Relationships, however, can also be hindered by negative professional beliefs and attitudes. These include stigmatising and prejudicial attitudes of some end-of-life practitioners towards people with severe mental illness, including those who are homeless (CERQual–high).^53–55,58,59,61–63,65^

Mental health staff feel that they have limited experience of caring for patients with severe mental illness at the end-of-life, and although some describe themselves as able to provide care others feel that they lack necessary expertise (CERQual–moderate).^53–55,58,64^ Some view end-of-life care as not mental health work, and are not interested in it or avoid doing it, whereas others embrace this (CERQual–moderate).^49,56,58,63,64^ Some mental health staff also describe end-of-life caring work as distressing and draining, and fear scrutiny when patients die (CERQual–moderate).^53–55,61,63^ There is recognition that staff caring for this group need support (ungraded–non research),^67^ and that the needs of those with severe mental illness at the end-of-life are not always met (ungraded–non research).^67,94^ End-of-life HCPs should become conversant with the needs of people with SMI and work closely with MH services (ungraded–non research).^67^

Education and training for all professional groups caring for people with severe mental illness at the end-of-life are important, and there is recognition that core preparation could be improved and greater opportunities provided for ongoing education (ungraded–non research).^67,68,74,77–79,92^ Professionals in both mental health and end-of-life services identify broad educational needs (CERQual–moderate)^46,53–55,57,61,63^ including in initial preparation (CERQual-very low).^49^ Some mental health nurses feel that that their preparation supports them in caring for people with severe mental illness during periods of physical illness and at the end-of-life, but others describe this being open to improvement (CERQual–moderate).^53–55,63^ In-service education for professionals to develop expertise in end-of-life care is recognised as limited, particularly in mental health care-providing organisations (ungraded–non research).^67,74,78,79,81,86^ Staff in both end-of-life and mental health services working together to improve knowledge and awareness through the establishment of link positions has been proposed (ungraded–non research),^67^ or through cross-training (CERQual–very low).^47,50^

#### Contexts of care

General practitioners act as gatekeepers to services and can both help and hinder access to palliative care and other services, and (in some instances) actively manage the palliative needs of people with severe mental illness within their practice (CERQual–moderate).^64,65^ No significant differences exist in the rates of ambulatory visits to GPs made by people with and without schizophrenia who are dying of cancer (Grade–very low).^10^ At the end-of-life people with severe mental illness visit different types of physician, as well as psychiatrists (CERQual–moderate)^49,64,65^ but people with schizophrenia are significantly less likely to visit a medical specialist before their deaths (Grade–very low).^10^ However initial contact is made there is recognition that support for people with mental health issues and approaching the end-of-life must be fast-tracked (ungraded–non research).^78^ Staff acknowledge that referrals are often complicated and lack information of a type which would promote communication between professionals, and with patients (CERQual–low).^57,58^ Following referral, skilled mental health assessment at the end-of-life is necessary to support care planning and the meeting of needs (ungraded–non research).^67^ Professionals in both services describe mental health assessments at the end-of-life as challenging, with staff needing help to deal with fears and uncertainties (CERQual–moderate).^56,58,61,65^

Psychiatrists and GPs both believe that people with severe mental illness are less likely than other groups to make use of specialist palliative care (CERQual–very low),^65^ although findings are inconsistent regarding utilisation rates of palliative care in the last year of life for those with schizophrenia in the community who have died from cancer (Grade–very low).^33,35,38^ No significant differences in rates of enrolment in hospices have been found for people with, and without, schizophrenia who have died of cancer (Grade–very low)^36,38^ although having depression ahead of a cancer diagnosis is associated with a significantly increased rate of hospice enrolment and length of stay (Grade–very low).^42^

People with severe mental illness at the end-of-life use long-stay, hospital, emergency departments and intensive care services in different ways from people without severe mental illness, but findings are inconsistent (Grade–very low).^10,33,35,40–42,44^ People with a schizophrenia diagnosis who have died of cancer have been found to have had significantly increased rates of use of long-term institutional care and to have had longer lengths of stay (Grade–very low).^10^ Studies also consistently report that people with severe mental illness who have died from cancer, heart failure, cirrhosis/liver disease or renal disease/dialysis are significantly less likely to have been admitted to hospital at the end-of-life (Grade-very low),^10,35,40,44^ but no differences have been noted for people dying from chronic lower respiratory disease (Grade–very low).^44^

People with severe mental illness at the end-of-life receive invasive interventions such as analgesia or opioid medication, chemotherapy and advanced diagnostic examinations in different ways, but research findings in this area are inconsistent (Grade–very low).^35,37,38,42^ People with schizophrenia who have died from cancer are significantly more likely to have had physician orders for life-sustaining treatment (Grade–very low).^36^ Veterans with pre-existing, but unspecified, mental health conditions where over half had a terminal condition of cancer or heart disease are more likely to receive care directed at controlling symptoms or supporting do not resuscitate orders (Grade–very low).^37^ In the UK end-of-life professionals report that standard guidance on resuscitation is lacking for people with severe mental illness (CERQual–very low),^65^ and end-of-life professionals in Australia report concerns over capacity to consent in relation to resuscitation orders for people with severe mental illness (CERQual–very low).^53–55^ No significant differences in the rates of cardiopulmonary resuscitation are found for those with and without severe mental illness at the end-of-life (Grade–very low).^36^

People with severe mental illness are known to have particular vulnerabilities arising from their mental health experiences, and their care at the end-of-life therefore requires a comprehensive team approach (ungraded–non research).^67,78,91^ Programmes and services require combinations of symptom relief and psychological, psychosocial and spiritual care (CERQual–high).^39,43,53–55,57,61,64^ Professionals report challenges in handling contact with the families of people at the end-of-life, especially where estrangements have occurred or where family members also have mental health issues (CERQual–moderate).^43,63,64^ In the case of veterans with PTSD at the end-of-life, families have been found to be as likely to receive a consultation regarding advanced care planning (ACP) as those without a diagnosis of PTSD (CERQual–very low).^39^ They also have unmet needs for emotional support and feel that their relatives were not treated with dignity and are dissatisfied with the care received (CERQual–very low).^45^

Having an advocate who is able to support a person with severe mental illness throughout their cancer journey and to prevent them from falling through the gaps in the care system, including at the end-of-life, is important (ungraded–non research).^67,68,73,74^

Professionals report that people with severe mental illness being referred to palliative care, and receiving services, appear to reflect the presence or absence of a strong advocate (CERQual–high).^39,43,53–55,57,61,64^ They are at risk of lacking advocacy to help navigate their end-of-life journeys, due to limited social and family support and, as a result, they can become ‘lost in the system’ (CERQual–high).^57,58,63,64^

Preferences for ACP at the end-of-life for people with severe mental illness are important, and means to support people to make their own decisions are needed even though accomplishing this can be difficult (ungraded–non research). ^68,77–79,85,91^ Professionals have concerns about discussing end-of-life preferences, fearing that symptoms of mental illness may influence understanding and expectations or that conversations may lead to further distress (CERQual–moderate).^57,59,63,64^ In the context of making decisions it is recognised as important not to assume that capacity is lacking (ungraded–non research).^57^ Professionals report discomfort determining patients’ capability to make health-related decisions, and tend to assume that mental capacity is lacking and as a result discussions around ACP are avoided (CERQual–moderate).^49,57,58^ Scenario-based research regarding ACP suggests that people with severe mental illness are able to designate end-of-life treatment preferences, and are open to discussing these (CERQual–moderate).^47,48,51,60^ People with severe mental illness are able to complete ACPs, but even with enabling legislation this rarely appears as standard practice (CERQual–moderate).^43,46,64^ Findings are inconsistent regarding the likelihood of people with a diagnosis of severe mental illness to have completed an advance directive (Grade–very low).^36^ A lack of confidence in open communications and experience in staff, especially when working with homeless people, possibly further explains the absence of ACPs for people with severe mental illness (CERQual–moderate).^43,57,59,62^

#### Living with severe mental illness

The provision of end-of-life care to people with severe mental illness can be challenged by patients’ behaviour associated with their mental health difficulties (ungraded–non research).^68,85^ Care complexities, including challenging behaviours, communication issues and side-effects from combined medications make addressing mental health issues at the end-of-life difficult (CERQual–moderate).^53–55,57,58,61,63,64^ Helpful factors include early referral to palliative care, enabling the building of trust and rapport (CERQual–moderate).^46,53–55,57,58,61,62,65^ Although people with severe mental illness often leave environments with which they are familiar at the end-of-life, staff can work together to support people without the need for moving (CERQual–moderate).^53–55,59,62,65^ This is harder for people who are also homeless and vulnerable, with deteriorations in physical health often leading to transfer to hospital where needs are poorly met (ungraded–non research).^68^ Examples exist of hostel staff trying to ensure that palliative care is provided in familiar environments for as long as possible (CERQual–moderate).^59,62^

Not being able to recognise their physical health needs, and the signs of deterioration, is a barrier to the receipt of end-of-life care (ungraded–non research).^67,68,73^ Practition-ers report people with severe mental illness as not always being able to recognise their declining health, and in a context of previous unsatisfactory health care encounters feel that they often present late to services (CERQual–moderate).^57,58,64,65^ The timely provision of palliative care can be hampered when people with severe mental illness (especially those who are homeless) are not recognised as approaching the end-of-life until late diagnosis of physical disease (CERQual–high).^57,59,64^ People who are homeless may be more concerned with day-to-day survival than with keeping appointments, challenging the identification of end-of-life trajectories and the provision of care (CERQual–moderate).^59,62^

## Discussion

Previous evidence reviews of end-of-life care for people with severe mental illness have either become very out-of-date,^12,14^ or have been much more limited in scope, than the review reported on here.^11,13,15^ With the purpose of informing future developments in all countries with developed health services which include systems of care for people with mental health needs, and systems of care for people who are dying, in this discussion we emphasise the implications of the evidence (as summarised in 10 of the 52 synthesis statements derived from both the descriptive quantitative and qualitative research) in which there is a high degree of confidence as assessed using the CERQual approach. As the summary statements assessed using the GRADE approach all attracted very low confidence these are not drawn on here.

### Partnership

First, reflecting recommendations from earlier reviews in this field,^11,12,16,19,20^ we highlight that formal and informal partnership opportunities should be taken and encouraged across the wider system of health and social care in order that the end-of-life needs of people with severe mental illness be better identified and met. Partnerships can involve representatives of mental health, end-of-life, primary care, social care and other services and are needed to promote information exchange and the integration of care. In a context in which there is often ‘no right place to die’ and in which mental health staff are often poorly equipped to care for people at the end-of-life, and where people with severe mental illness face frequent moves between services, finding ways of supporting people to die in the locations of their choice is a priority. Although we confirm the observation has been made before that end-of-life and mental health services may share some similarities in terms of their treatment philosophies,^12,20^ we concur with the findings of the scoping review by Relyea et al.^13^ that still missing from research in this area are studies examining specific approaches to providing more collaborative care.

Building trust at the end-of-life is an important goal,^95^ but in the area of professional practice a significant finding in which a high degree of confidence holds is that staff can find it difficult to invest in relationships with people with severe mental illness at the end-of-life due to the upset caused when patients are transferred to other facilities. However, what this review highlights is that mental health staff, many of whom find talking about death and dying with patients difficult, find that when opportunities are found patients are receptive. Knippenberg et al.^96^ report that patients with severe mental illness and additional severe physical health issues do not routinely speak, or think, about end-of-life issues but when probed by researchers they did then discuss the terminal phase of life. In the wider context of the coronavirus pandemic we observe how mental health staff are increasingly being exposed to dying and death, leading to important new guidance for professionals and their support in this area.^97^

It has long been acknowledged that stigma and prejudicial attitudes are experienced by those living with severe mental illness,^98^ reflected in the recent launching of a *Lancet* commission in this area.^99^ Supporting other work in this area^13,95,100,101^ this review has shown that stigma and discrimination remain major problems for people with severe mental illness at the end-of-life, especially for those who are homeless. In the case of staff working in end-of-life services stigmatising and prejudicial attitudes towards people with severe mental illness, and particularly people who are homeless, can affect decision-making. This speaks clearly to the need for education, support and supervision.

What this review has demonstrated with a high degree of confidence is that programmes and services for people at the end-of-life require a comprehensive team approach incorporating symptom relief, psychological and psychosocial support and spiritual care. Comprehensive services of this type are exactly as should be expected by all members of the population,^12,14^ but the importance of this for people with severe mental illness at the end-of-life is worth restating for the purposes of promoting parity of esteem. Whilst parity is an international goal,^3^ even when research and other evidence in this area is presented for use by policymakers it typically neglects to address end-of-life care specifically.^98^ A practical strategy to promote comprehensive care in which a high level of confidence exists is the use of capable advocates, able to increase the referral of people with severe mental illness to palliative care services, and to help make sure palliative care is provided and received. Not having an advocate risks people with severe mental illness lacking social and family support becoming ‘lost in the system’. Taken together, these synthesis summaries have important implications for the identification of roles for members of the care team in coordinating services across boundaries, advocating for and on behalf of patients, and providing direct care. More generally, avoiding being lost to support means strong care coordination, reflecting findings from other studies involving the organisation of services for people with severe mental illness.^102^

Finally, this review found that the timely provision of end-of-life care to people with severe mental illness, and particularly those who are homeless, is hindered by the problem of delayed diagnoses. This diagnostic overshadowing is an ongoing problem that has been described in the literature for well over a decade.^11,12,14^ This reinforces the need for more proactive, routine, physical health care as high-quality palliative care is an international human right to which all should be able to have access without delay.^103–105^

## Limitations

All the research studies included in this review were undertaken in high income countries with developed health systems, and none evaluated interventions to improve care. The lack of consensus surrounding the term ‘severe mental illness’ presented itself as a challenge for database and related searching, and even with the help of knowledgeable stakeholders our need to set parameters for other searches means that some material may not have been included. It is also recognised that the search for policy and guidance from the UK only (reflecting the particular interests of the project’s funder), rather than extending this to other countries around the world including those with broadly comparable health systems, represents a limitation. With only English-language items included the possibility also exists that important research and other evidence has been missed.

## Conclusion

This rigorous, mixed methods, systematic review and thematic synthesis has brought together research from 10 countries, plus exemplar policy and guidance from the four nations of the UK, in an important but neglected area. Beyond people with severe mental illness, findings have relevance for the end-of-life care of other disadvantaged groups for whom health inequalities persist. With regards to future work, end-of-life care for people with severe mental illness is a wide-open area for well-designed research, including intervention studies of which no examples were found meeting the inclusion criteria for this review. Studies are needed examining the experiences of people and their carers with severe mental illness at the end-of-life, along with studies co-producing, introducing and evaluating new ways of providing and organising care. This programme of research should also include projects focusing on particularly disadvantaged groups, including people with severe mental illness at the end-of-life who are also homeless or who are in prison. Candidate interventions include advanced planning, advocacy and improved education for professionals along with the development of new or enhanced roles for practitioners and the introduction of models of integrated provision spanning the mental health, end-of-life and related care systems.

## Supplemental Material

sj-pdf-1-pmj-10.1177_02692163211037480 – Supplemental material for End of life care for people with severe mental illness: Mixed methods systematic review and thematic synthesis (the MENLOC study) Click here for additional data file. Supplemental material, sj-pdf-1-pmj-10.1177_02692163211037480 for End of life care for people with severe mental illness: Mixed methods systematic review and thematic synthesis (the MENLOC study) by Deborah Edwards, Sally Anstey, Michael Coffey, Paul Gill, Mala Mann, Alan Meudell and Ben Hannigan in Palliative Medicine

sj-pdf-2-pmj-10.1177_02692163211037480 – Supplemental material for End of life care for people with severe mental illness: Mixed methods systematic review and thematic synthesis (the MENLOC study) Click here for additional data file. Supplemental material, sj-pdf-2-pmj-10.1177_02692163211037480 for End of life care for people with severe mental illness: Mixed methods systematic review and thematic synthesis (the MENLOC study) by Deborah Edwards, Sally Anstey, Michael Coffey, Paul Gill, Mala Mann, Alan Meudell and Ben Hannigan in Palliative Medicine

sj-pdf-3-pmj-10.1177_02692163211037480 – Supplemental material for End of life care for people with severe mental illness: Mixed methods systematic review and thematic synthesis (the MENLOC study) Click here for additional data file. Supplemental material, sj-pdf-3-pmj-10.1177_02692163211037480 for End of life care for people with severe mental illness: Mixed methods systematic review and thematic synthesis (the MENLOC study) by Deborah Edwards, Sally Anstey, Michael Coffey, Paul Gill, Mala Mann, Alan Meudell and Ben Hannigan in Palliative Medicine

sj-pdf-4-pmj-10.1177_02692163211037480 – Supplemental material for End of life care for people with severe mental illness: Mixed methods systematic review and thematic synthesis (the MENLOC study) Click here for additional data file. Supplemental material, sj-pdf-4-pmj-10.1177_02692163211037480 for End of life care for people with severe mental illness: Mixed methods systematic review and thematic synthesis (the MENLOC study) by Deborah Edwards, Sally Anstey, Michael Coffey, Paul Gill, Mala Mann, Alan Meudell and Ben Hannigan in Palliative Medicine

sj-pdf-5-pmj-10.1177_02692163211037480 – Supplemental material for End of life care for people with severe mental illness: Mixed methods systematic review and thematic synthesis (the MENLOC study) Click here for additional data file. Supplemental material, sj-pdf-5-pmj-10.1177_02692163211037480 for End of life care for people with severe mental illness: Mixed methods systematic review and thematic synthesis (the MENLOC study) by Deborah Edwards, Sally Anstey, Michael Coffey, Paul Gill, Mala Mann, Alan Meudell and Ben Hannigan in Palliative Medicine
