# Supplementary material for: End of life care for people with severe mental illness: Mixed methods systematic review and thematic synthesis (the MENLOC study)
Source: Palliat Med. 2021 Sep 3;35(10):1747–60. doi: 10.1177/02692163211037480 (PMC8637363; doi:10.1177/02692163211037480)
Supplement: sj-pdf-1-pmj-10.1177_02692163211037480 – Supplemental material for End of life care for people with severe mental illness: Mixed methods systematic review and thematic synthesis (the MENLOC study) [file sj-pdf-1-pmj-10.1177_02692163211037480.pdf]

Supplementary file 1: Ovid MEDLINE (R) final search strategy

<1946 to December 06, 2018> and updated December 2019

1 exp Palliative care/ (49655)

2 exp Hospice care/ (5949)

3 exp Terminal Care/ (48040)

4 exp Terminally ill/ (6227)

5 ("palliative care" or hospice or "end of life care" or end-of-life).tw. (43896)

6 ((hospice or terminal*) adj3 (care or caring or ill*)).tw. (12766)

7 ("Irreversible condition" or "terminal condition" or fatal illness).tw. (791)

8 ("last year of life" or LYOL or "end of life" or "end of their lives" or "last six months of life" or "last 6 months of life").tw. (19924)

9 (end-stage disease* or end stage disease* or end-stage ill* or end stage ill* or end-stage or end stage).tw. (60633)

10 (expected adj3 die).tw. (215)

11 (imminent adj3 death).tw. (561)

12 ("Dying soon" or "expected death" or "imminently dying" or Moribund).tw. (2493)

13 conservative treatment/ (1401)

14 (conservative adj2 (treatment or management)).tw. (42831)

15 Withholding Treatment/ (10909)

16 Treatment Refusal/ (11477)

17 (Refus* adj3 (treat* or care or intervention or dialysis)).tw. (4855)

18 ((withdrew or withdraw* or withhold*) adj3 (treat* or car* or intervene* or therap* or dialysis or transplant*)).tw. (15455)

19 or/1-18 (244771)

20 exp Neoplasms/ (3107101)

21 (cancer* or tumo?r* or neoplas* or malignan* or carcinoma* or adenocarcinoma* or adeno?carcinoma* or choriocrcinoma* or leukemia* or leukaemia* or metastat* or sarcoma* or teratoma* or lymphoma).tw. (3243976)

22 ("Enzymatic disease*" or "enzyme disease*").tw. (25)

23 Genetic Disease, Inborn/ (13207)

24 ("duchenne muscular dystrophy" or "amyotrophic lateral sclerosis" or ALS).tw. (40207)

25 amyotrophic lateral sclerosis/ (16921)

26 Muscular dystrophy, Duchenne/ (4706)

27 ("Genetic disease" or "genetic condition").tw. (7305)

28 Cystic Fibrosis/ (33150)

29 ("cystis fibrosis" or CF).tw. (36968)

30 exp Multiple Organ Failure/ (10209)

31 ("Organ failure" or "chronic organ failure").tw. (17137)

32 Pulmonary Disease, Chronic Obstructive/ (34379)

33 ("renal insufficiency" or "serious physical illness").tw. (21134)

34 (Chronic obstructive pulmonary disease or COPD).tw. (54881)

35 (chronic adj3 (illness or condition or disease*)).tw. (250692)

36 "chronic medical condition".tw. (382)

37 Heart Failure/ (107446)

38 Renal Insufficiency/ (14803)

39 Liver Failure/ (6828)

40 (chronic adj2 ("cardiac failure" or "liver failure" or "kidney failure" or "end-stage renal disease" or ESRD or "renal failure" or "heart failure")).tw. (44143)

41 Chronic Disease/ (251517)

42 Health service utilization.mp. (1569)

43 lifestyle-related factor*.mp. (354)

44 or/20-43 (4717135)

45 exp Bipolar Disorder/ (37764)

46 exp Schizophrenia/ (99011)

47 (schizo* or "mood disorder*" or "personality disorder*" or psychotic* or psychosis or psychoses).tw. (197049)

48 (bipolar or mania or Schizophrenia).tw. (151754)

49 (Depression adj2 (psychosis or psychotic or severe or major)).tw. (28840)

50 exp Mental Disorders/ (1143274)

51 mental health condition*.tw. (1954)

52 exp Personality Disorders/ (39433)

53 exp psychotic disorders/ (48805)

54 exp schizoaffective disorder/ (48805)

55 exp psychosis/ (48805)

56 Paranoid Disorders/ (3987)

57 ("severe mental illness" or "persistent mental illness").tw. (3969)

58 ((chronic* or sever* or serious or persistent* or enduring or debilitating) adj2 (mental* or psychological*) adj2 (ill* or disorder* or health)).tw. (12556)

59 exp Mentally Ill Persons/ (5926)

60 (SPMI or SMI).tw. (3830)

61 or/45-60 (1239759)

62 (dementia or Alzheimer).ti. (52380)

63 (bipolar electrocoagulation or bipolar radiofrequency or bipolar tumour probe or bipolar diathermy).tw. (1123)

64 ("respiratory depression" or "marrow depression" or "hematologic* depression").tw. (6399)

65 (child* or adoles* or pediatric or paediatric).tw. (1520041)

66 (Algeria$ or Egypt$ or Liby$ or Morocc$ or Tunisia$ or Western Sahara$ or Angola$ or Benin or Botswana$ or Burkina Faso or Burundi or Cameroon or Cape Verde or Central African Republic or Chad or Comoros or Congo or Djibouti or Eritrea or Ethiopia$ or Gabon or Gambia$ or Ghana or Guinea or Keny$ or Lesotho or Liberia or Madagasca$ or Malawi or Mali or Mauritania or Mauritius or Mayotte or Mozambiq$ or Namibia$ or Niger or Nigeria$ or Reunion or Rwand$ or Saint Helena or Senegal or Seychelles or Sierra Leone or Somalia or South Africa$ or Sudan or Swaziland or Tanzania or Togo or Ugand$ or Zambia$ or Zimbabw$ or China or Chinese or Hong Kong or Macao or Mongolia$ or Taiwan$ or Belarus or Moldov$ or Russia$ or Ukraine or Afghanistan or Armenia$ or Azerbaijan or Bahrain or Cyprus or Cypriot or Georgia$ or Iran$ or Iraq$ or Jordan$ or Kazakhstan or

Kuwait or Kyrgyzstan or Leban$ or Oman or Pakistan$ or Palestin$ or Qatar or Saudi Arabia or Syria$ or Tajikistan or Turkmenistan or United Arab Emirates or Uzbekistan or Yemen or Bangladesh$ or Bhutan or British Indian Ocean Territory or Brunei Darussalam or Cambodia$ or India$ or Indonesia$ or Lao or People's Democratic Republic or Malaysia$ or Maldives or Myanmar or Nepal or Philippin$ or Singapore or Sri Lanka or Thai$ or Timor Leste or Vietnam or Albania$ or Andorra or Bosnia$ or Herzegovina$ or Bulgaria$ or Croatia$ or Faroe Islands or Greenland or Liechtenstein or Lithuani$ or Macedonia or Malta or maltese or Romania or Serbia$ or Montenegro or Svalbard or Argentina$ or Belize or Bolivia$ or Brazil$ or Chilean or Colombia$ or Costa Rica$ or Cuba or Ecuador or El Salvador or French Guiana or Guatemala$ or Guyana or Haiti or Honduras or Jamaica$ or Nicaragua$ or Panama or Paraguay or Peru or Puerto Rico or Suriname or Uruguay or Venezuela or developing countr$ or south America$).ti,sh. (1225437)

67 Academic Dissertations/ (0)

68 thesis.tw. (7167)

69 book.pt. (0)

70 Books/ (3174)

71 or/62-70 (2653676)

72 19 and 44 and 61 (2434)

73 72 not 71 (2126)

74 limit 73 to (english language and humans and "all adult (19 plus years)") (1217)
