# Supplementary material for: End of life care for people with severe mental illness: Mixed methods systematic review and thematic synthesis (the MENLOC study)
Source: Palliat Med. 2021 Sep 3;35(10):1747–60. doi: 10.1177/02692163211037480 (PMC8637363; doi:10.1177/02692163211037480)
Supplement: sj-pdf-2-pmj-10.1177_02692163211037480 – Supplemental material for End of life care for people with severe mental illness: Mixed methods systematic review and thematic synthesis (the MENLOC study) [file sj-pdf-2-pmj-10.1177_02692163211037480.pdf]

Table of included grey literature

**Author**<br>**Title**<br>**url**	**Focus**
1. Addicott and Ashton 2010[61]<br>Delivering better care at the end of life: The next steps. The King's Fund<br>https://www.kingsfund.org.uk/sites/default/files/Delivering-better-care-end-of-life-Kings-Fund-January-2010-Leeds-Castle-EoLC.pdf	EoLC<br>Some sections on EoLC for people with pre-existing SMI
2. Care Quality Commission 2016[62]<br>People with a mental health condition: A different ending: Addressing inequalities in end of life care<br>https://www.cqc.org.uk/sites/default/files/20160505%20CQC_EoLC_MentalHealth_FINAL_2.pdf	EoLC and MH<br>Includes information on EoLC for those with pre-existing SMI
3. Care Quality Commission 2016[62]<br>A different ending: Addressing inequalities in end of life care<br>https://www.cqc.org.uk/sites/default/files/20160505%20CQC_EoLC_OVERVIEW_FINAL_3.pdf	EoLC<br>Some sections on EoLC for people with pre-existing SMI
4. Care Quality Commission 2017[63]<br>A second class ending: Exploring the barriers and championing outstanding end of life care for people who are homeless<br>https://www.cqc.org.uk/sites/default/files/20171031_a_second_class_ending.pdf	EoLC<br>Some sections on EoLC for people with pre-existing SMI
5.Department of Health 2008[64]<br>End of life care strategy. promoting high quality care for all adults at the end of life<br>https://assets.publishing.service.gov.uk/government/uploads/system/uploads/attachment_data/file/136431/End_of_life_strategy.pdf	EoLC<br>Some sections on EoLC for people with pre-existing SMI
6. Department of Health 2016[65]<br>Health and wellbeing 2026. Delivering together<br>https://www.health-ni.gov.uk/sites/default/files/publications/health/health-and-wellbeing-2026-delivering-together.pdf	General health care<br>Some sections on mental health and physical health
7. Department of Health 2018[66]<br>Service framework for mental health and wellbeing 2018-2021<br>https://www.health-ni.gov.uk/sites/default/files/consultations/health/MHSF%20-%20Service%20Framework%20-%202018-2021.PDF	MH<br>Some sections on EoLC for people with pre-existing SMI

8. General Medical Council 2010[15]<br>Treatment and care towards the end of life: Good practice in decision making<br>https://www.gmc-uk.org/-/media/documents/treatment-and-care-towards-the-end-of-life---english-1015_pdf-48902105.pdf	EoLC<br>Some sections on EoLC for people with pre-existing SMI
9. Help the Hospices 2006[67]<br>Hospice and palliative care –Access for all<br>https://www.issuelab.org/resources/17279/17279.pdf	EoLC<br>Some sections on EoLC for people with pre-existing SMI
10. Independent Cancer Taskforce 2015[16]<br>Achieving world-class cancer outcomes. A strategy for England 2015-2020.<br>https://www.cancerresearchuk.org/sites/default/files/achieving_world-class_cancer_outcomes_-_a_strategy_for_england_2015-2020.pdf	Cancer<br>Some sections on EoLC for people with pre-existing SMI
11. Leadership Alliance for the Care of Dying People 2014[68]<br>One chance to get it right: Improving people's experience of care in the last few days and hours of life<br>http://wales.pallcare.info/files/One_chance_to_get_it_right.pdf	EoLC<br>Some sections on EoLC for people with pre-existing SMI
12. Marie Curie 2016[69]<br>Marie Curie response: Mental health strategy for Scotland<br>https://www.mariecurie.org.uk/globalassets/media/documents/policy/briefings-consultations/scotland-briefings/marie-curie-response-mental-health-strategy.pdf	EoLC and MH<br>Includes information on EoLC for those with pre-existing SMI
13. Marie Curie 2016[70]<br>Marie Curie briefing: Mental health and terminal illness<br>https://www.mariecurie.org.uk/globalassets/media/documents/policy/policy-publications/october-2016/marie-curie-briefing-mental-health.pdf	EoLC and MH<br>Includes information on EoLC for those with pre-existing SMI
14. Marie Curie 2017[71]<br>Marie Curie briefing: Inequities in palliative care – Mental health<br>https://www.mariecurie.org.uk/globalassets/media/documents/policy/briefings-consultations/scotland-briefings/marie-curie-briefing-inequities-mental-health.pdf	EoLC and MH<br>Includes information on EoLC for those with pre-existing SMI
15. Addington-Hall 2000[72]<br>Positive partnerships: Palliative care for adults with severe mental health problems. Occasional Paper 17<br>https://www.palliativecarescotland.org.uk/content/publications/PalliativeCareforAdultswithSevereMentalHealthProblems.pdf	EoLC and MH<br>Includes information on EoLC for those with pre-existing SMI
16. National Institute of Clinical Excellence 2004[73]<br>Guidance on cancer services improving supportive and palliative care for adults with cancer	Cancer<br>Some sections on EoLC for people with pre-existing SMI

https://www.nice.org.uk/guidance/csg4/resources/improving-supportive-and-palliative-care-for-adults-with-cancer-pdf-773375005	
17. NHS England 2019[74]<br>The NHS long term plan<br>https://www.longtermplan.nhs.uk/publication/nhs-long-term-plan/	General health care<br>Some sections on EoLC or the care of people with SMI
18. NHS England 2014[75]<br>Five year forward view.<br>https://www.england.nhs.uk/wp-content/uploads/2014/10/5yfv-web.pdf	General health care<br>Some sections on EoLC for people with MH conditions
19. NHS England 2017[76]<br>Next steps on the NHS five year forward view<br>https://www.england.nhs.uk/publication/next-steps-on-the-nhs-five-year-forward-view/	General health care<br>Some sections on EoLC for people with pre-existing SMI
20. NHS Lothian 2010[77]<br>Living and dying well in Lothian<br>https://www.nhslothian.scot.nhs.uk/OurOrganisation/Strategies/ladwinlothian/Documents/Palliative%20Care%20Strategy%202010%20-15%20VER%2023%20FINAL.pd	EoLC<br>Some sections on EoLC for people with pre-existing SMI
21. Royal College of Physicians 2007[78]<br>Palliative care services: Meeting the needs of patients. Report of a working party 2007<br>https://cdn.shopify.com/s/files/1/0924/4392/files/palliative-care-services.pdf?15599436013786148553	EoLC<br>Some sections on EoLC for people with pre-existing SMI
22. Scottish Government 2017[79]<br>Mental health strategy: 2017-2027<br>https://www2.gov.scot/resource/0039/00398762.pdf	MH<br>Some sections on EoLC for people with pre-existing SMI
23. Social Care Institute for Excellence 2013[80]<br>Dying well at home: Research evidence<br>https://www.scie.org.uk/publications/guides/guide48/files/guide48_researchevidence.pdf	EoLC<br>Some sections on EoLC for people with pre-existing SMI
24. Welsh Government 2012[81]<br>Together for mental health: A strategy for mental health and wellbeing in Wales<br>https://gov.wales/docs/dhss/publications/121031tmhfinalen.pdf	MH<br>Some sections on EoLC for people with pre-existing SMI
25. Welsh Government 2018[82]<br>A healthier Wales: Our plan for health and social care<br>https://www.basw.co.uk/system/files/resources/180608healthier-wales-mainen.pdf	General health care<br>Some sections on EoLC or the care of people with mental ill health
26. Worldwide Palliative Care Alliance 2017[83]	EoLC<br>Some sections on EoLC for people with pre-existing SMI

Building integrated palliative care programs and services<br>http://www.thewhpca.org/resources/category/building-integrated-palliative-care-programs-and-services	
27. Healthcare Quality Improvement Partnership 2019[84]<br>National audit of care at the end of life. First round of the audit (2018/19) report England and Wales<br>https://www.hqip.org.uk/wp-content/uploads/2019/07/National-Audit-of-Care-at-the-End-of-Life-	EoLC<br>A section on mental health providers
28. Kennedy et al 2013[85]<br>Homelessness and end of life care. Practical information and tools to support the needs of homeless people who are approaching the end of life, and those who are bereaved<br>https://www.mungos.org/publication/homelessness-palliative-care/	EoLC<br>Some sections on EoLC for people with pre-existing SMI

Key: EoLC: end of life care; MH: mental health; SMI: severe mental illness

Characteristics of included retrospective cohort studies

**Author, Year, Country**<br><br>**Aim**<br>**Mental health diagnosis**<br>**Terminal / Chronic condition or cause of death**<br>**Definition of EoL/ PC**	**Setting**<br>**Patient characteristics**	**Design**<br>**Data sources**<br>**Outcomes**<br>**Outcome measures**
Study 1<br><br>Butler and O'Brien 2018[26]<br>New Zealand<br><br>Aim<br>To compare the rate of access to PC services for people with SPMI with the rate for the general population<br><br>MH diagnosis<br>SPMI<br><br>Terminal condition<br>Not linked to a specific terminal condition<br><br>Definition of EoL/ PC<br>SPC services in the LYoL	Setting<br>SPC services within a district public health board<br><br>Participants<br>Decedents with SPMI utilising SPC services (n=147)<br>Decedents from the general population utilising PC services (n=23,0571)<br><br>Age (years) Mean$\pm$SD<br>SMPI Cohort<br>18-24 (2%); 25-44 (16%); 45-64 (42%); 65-84 (31%); 85+ (9%)<br><br>General Population<br>18-24 (19%); 25-44 (36%); 45-64 (30%); 65-84 (13%); 85+ (2%)<br><br>Gender<br>SMPI Cohort: Male (78%)<br>General Population: Male (47.5%)<br><br>Ethnicity<br>SPMI Cohort<br>European (79%); Maori (15%)<br>Pacific (3%); Asian (2%)<br>General Population<br>European (65.5%); Maori (12.5%)	Design<br>Retrospective cohort (over 7 years)<br><br>Data sources<br>The MH data came from the Programme for the Integration of MH Data, which is a national MH and addiction database of service activity and outcomes. The information on this database is collected from district health boards and non-governmental organizations Population information came from Ministry of Health reports and reported data limited to the Capital and Coast District Health Board region<br><br>Outcomes<br>Rate of access to PC services<br>The rate ratio of SPC utilization was calculated by dividing the rate of contacts for people with SPMI by the rate of contacts for the general population<br><br>Outcome measures<br>The number of SPC service contacts in the LYoL for people with SPMI was compared with the number of SPC service contacts within the general population

	Pacific (8%); Asian (14%)	
Study 2<br><br>Fond et al 2019[29]<br>France<br><br><u>Aim</u><br>To establish whether the EoLC delivered to patients with schizophrenia and cancer differed from that delivered to patients with cancer who did not have a diagnosis of mental illness<br><br><u>MH diagnosis</u><br>Schizophrenia<br><br><u>Causes of death</u><br>Brain cancer, liver cancer, or any metastatic solid cancer.<br><br><u>Definition of EoLC/PC</u><br>Last 31 days of life	<u>Setting</u><br>French hospital care<br><br><u>Participants</u><br>Decedents with schizophrenia<br><br><u>Age (years)</u><br>Matched cohort<br>15–62 (22.2%)<br>63–71 (24.1%)<br>72–81 (28.4%)<br>≥82 (25.3%)<br><br>Schizophrenia cohort<br>15-62 (48.6%)<br>63–71 (24.7%)<br>72–81 (17.3%)<br>≥82 (9.4%)<br><br><u>Gender</u><br>Matched cohort: Male (58%)<br>Schizophrenia cohort: Male (56%)<br><br><u>Ethnicity</u><br>No data reported	<u>Design</u><br>Retrospective population-based cohort study (over 4 years)<br><br><u>Data sources</u><br>Data from Programme de Médicalisation des Systèmes d'Information, the French national hospital database in which administrative & medical data are systematically collected for acute & psychiatric care.<br><br><u>Outcomes</u><br>Health service utilization in 6 months prior to death for people with schizophrenia and cancer compared to those with cancer only and no schizophrenia diagnosis<br><br><u>Outcome measures</u><br>Access to PC in last 31 days of life and last 3 days of life chemotherapy in last 14 days of life<br><br>Within the last 31 days of life<br>Artificial nutrition (ie, enteral or parenteral nutrition)<br>Tracheal intubation, mechanical ventilation<br>Gastrostomy (or change in gastrostomy status)<br>Cardiopulmonary resuscitation<br>Dialysis, Blood transfusion, Surgery<br>Imaging. Endoscopy<br>At least one ED admission<br>At least one ICU admission<br>At least one air extraction chamber (ie, sterile chamber) admission<br>More than one admission to acute care unit<br>Length of stay in acute care unit in<br>Death in ED ICU
Study 3<br><br>Ganzini et al 2010[30]<br>USA<br><br><u>Aim</u>	<u>Setting</u><br>VA Medical centres within Northwest United States (Oregon, Washington, and Alaska)<br><br><u>Participants</u><br>Veterans / decedents with schizophrenia or schizoaffective disorder who died of cancer (n=60) were compared veterans / decedents	<u>Design</u><br>Retrospective cohort (over 6 years)<br><br><u>Data sources</u><br>Data from the data management system of the U.S. Department of Veterans Affairs Northwest Health Network–Veterans Integrated Service Network 20<br>Was obtained along with medical record review

To compare measures of quality of EoLC among veterans with and without schizophrenia who died of cancer<br><br><u>MH diagnosis</u><br>Schizophrenia or schizoaffective disorder<br><br><u>Cause of death</u><br>Cancer<br><br><u>Definition of EoL/ PC</u><br>Last 6 months of life	with no major mental illness who died of cancer (n=196)<br><br><u>Age (years)</u> Mean+SD<br>Cancer and Schizophrenia: 64.1±11.5<br>Cancer without Schizophrenia: 70.5±10.4<br><br><u>Gender</u><br>Cancer and Schizophrenia: Male (93%)<br>Cancer without Schizophrenia: Male (97%)<br><br><u>Ethnicity</u><br>No data reported	<u>Outcomes</u><br>Quality of EoLC<br><br><u>Outcome measures</u><br>Enrolment in and length of hospice care<br>Prescription of any opioids in the last six months of life but before hospice enrolment<br>Presence of an AD or physician orders for
Study 4<br><br>Huang et al 2017[31]<br>USA<br><br><u>Aim</u><br>To gain knowledge on MH disparities in EoLC<br>To identify at least three quality indicators of EoLC processes that are associated with MH diagnoses<br><br><u>MH diagnosis</u><br>Any MH diagnosis – not specified<br><br><u>Terminal conditions</u><br>Cancer (30.1%) , Heart disease (20.1%)<br>Other (not reported)<br><br><u>Definition of EoL/ PC</u><br>Processes of care at EoL<br>Timeframe not specified	<u>Setting</u><br>Not reported<br><br><u>Participants</u><br>Inpatient veterans / decedents (n=5476)<br><br><u>Age (years)</u> Mean+SD<br>70.1+22.5<br><br><u>Gender</u><br>Male (98%)<br><br><u>Ethnicity</u><br>White (65%)<br>Black (34%)	<u>Design</u><br>Retrospective cohort (over 7 years)<br><br><u>Data sources</u><br>Data were drawn from the Best Practices for EoLC for Our Nation's Veterans (BEACON) trial conducted<br><br><u>Outcomes</u><br>Quality of EoLC<br><br><u>Outcome measures</u><br>Opioid order, do not resuscitate order, intravenous line infusing at time of death, nasogastric tube, and physical restraints
Study 5<br><br>Huang et al 2018[32]<br>Taiwan	<u>Setting</u><br>All medical care covered by the National Health Insurance program which covers	<u>Design</u><br>Retrospective cohort (over 12 years)<br><br><u>Data sources</u>

<u>Aim</u><br>To compare the medical treatments received by cancer patients with and without schizophrenia within 1 and 3 months prior to their death<br><br><u>MH diagnosis</u><br>Schizophrenia<br><br><u>Terminal conditions</u><br>Cancer<br>(breast, lung, liver, colorectal, oral, prostrate)<br><br><u>Definition of EoL/ PC</u><br>Within 1 month or 3 months of death	approximately 99% of the population and has contracts with 97% of the hospitals and clinics<br><br><u>Participants</u><br>Patients from 20 years old who were newly diagnosed as having one of six common cancers between 2000 and 2012 cancer but who were not alive by the end of the study period<br>Matched (ratio 1:4) sample decedents with no previous schizophrenia diagnoses (n=7644)<br><br><u>Age (years)</u><br><50 (23.4%), 50-69 (44.8%), >70 (31.7%)<br><br><u>Gender</u><br>Males (55%)<br><br><u>Ethnicity</u><br>Not reported	Nationwide population-based cohort study based on the National Health Insurance Research Database of Taiwan<br><br><u>Outcomes</u><br>Medical treatments or interventions 1-3 prior to their death<br>Utilization of medical care and PC 1-3 months prior to their death<br>Advanced diagnostic examinations 1-3 months prior to their death<br><br><u>Outcome measures</u><br>Numbers receiving chemotherapy, invasive intervention and advanced diagnostic examination<br>Number of inpatient days<br>ICU utilisation (days)<br>PC and hospice ward care utilization (days)<br>PC consultation and hospice home care
Study 6<br><br>Lavin et al 2017[34]<br>USA<br><br><u>Aim</u><br>To examine whether having a psychiatric illness is associated with site of death or health care utilization in the last month of life<br><br><u>MH diagnosis</u><br>Psychiatric illness ((ICD)-9 diagnosis of mood, anxiety, and/or psychotic disorder or prescription medications for psychiatric illness prevalence 10.6% (5.6% based on documented diagnosis and 5.0% based on psychiatric medication prescription)	<u>Setting</u><br>UW Medicine health care system, an integrated health care system that includes four diverse academic and community hospitals)<br><br><u>Participants</u><br>Decedents without psychiatric illness (n= 14,499) and decedents with psychiatric illness (n= 1715)<br><br><u>Age (years)</u> Mean±SD<br>Psychiatric Illness :61.3± 14.1<br>No Psychiatric Illness: 64.6±12.3<br><br><u>Gender</u><br>Psychiatric Illness: Male (50.3%)<br>No Psychiatric Illness: Male (53.8%)	<u>Design</u><br>Retrospective cohort (over 4 years)<br><br><u>Data collection methods</u><br>Data were extracted from UW Medicine electronic health records and from Washington State death certificates.<br><br><u>Outcomes</u><br>Occurrence and length of nonsurgical acute care hospitalizations;<br>Occurrence and number of ED visits that did not result in a subsequent nonsurgical hospitalization<br>Occurrence and length of ICU stays;<br>Place of death

Chronic conditions (of relevance)<br>Nonhematologic cancer, chronic obstructive pulmonary disease, congestive heart failure, chronic liver disease, chronic renal disease<br><br>Definition of EoL/ PC<br>Last month of EoL	Ethnicity (psychiatric illness cohort)<br>Hispanic ethnicity (1.7%)<br>White (84.3%); Black (7.4%)<br>Native American (2.2%); Asian (3.3%)<br>Pacific islander (0.2%); Other race (1.4%)<br>Mixed race (1.2%)	
Study 7<br><br>Martens et al 2013[35]<br>Chochinov et al 2012[28]<br><br>Canada<br><br>Aim<br>To compare place and cause of death with and without schizophrenia in Manitoba, Canada[35]<br><br>To compare rate of health care services, including PC, used in the last 6–24 months of life for patients with and without schizophrenia[28]<br><br>MH diagnosis<br>Schizophrenia<br><br>Cause of death (of relevance)<br>Cancer<br><br>Definition of EoL/ PC<br>PC used in 6–24 months of life	Setting<br>All hospital and community-based PC within the universal healthcare system for the province of Manitoba<br><br>Participants<br>Decedents with schizophrenia diagnoses in 12 years prior to death (n=3943)<br>Matched (ratio 1:3) decedents with no previous schizophrenia diagnoses (n=11,827)<br><br>Age (years) Mean±SD (All)<br>73.4 years SD=16.5<br><br>Gender (All)<br>Male (43.3%)<br><br>Ethnicity<br>Not reported	Design<br>Retrospective cohort (over 12 years)<br><br>Data sources<br>De-identified ('anonymised') administrative claims data were used from the Population Health Research Data Repository housed at the Manitoba Centre for Health Policy, University of Manitoba<br><br>Outcomes<br>Relationship between place of death by cause of death[35]<br>Health service use patterns by place of death in last 6 months of life[35]<br>Health service utilization rates in the six months prior to death for decedents with and without schizophrenia[28]<br><br>Outcome measures<br>Place of death – hospital, home, nursing home and other[35]<br>GP visit rate per person[35]<br>Physician specialist visit rate per person[35]<br>Inpatient hospital separation rates per person[35]<br>Rate of inpatient hospital days per person[35]<br>Visits to GPs (family practice physician), specialists, inpatient stays in acute care hospitals, use of nursing homes (long-term institutional care, also referred to as personal care homes)[28]<br>Use of PC[28]<br>Use of home care services[28]<br>Utilization of analgesics[28]
Study 8<br><br>McDermott et al 2018[36]<br>USA	Setting<br>Acute healthcare services and hospice care within the USA<br><br>Participants	Design<br>Retrospective cohort (over 3 years)<br><br>Data sources

Aim<br>To assess the relationship between depression and health care utilization at EoL among older adults (ages >=67) diagnosed with advanced non-small cell lung cancer from 2009 to 2011<br><br>MH diagnosis<br>Pre cancer, diagnosis-time or post diagnosis depression<br><br>Terminal condition<br>Advanced non-small cell lung cancer<br><br>Definition of EoL/ PC<br>Last 30 days of life	Decedents with:<br>Pre-cancer depression (n=1485)<br>Diagnosis-time depression (n= 709)<br>Post diagnosis depression (n= 1189)<br>No depression at any time (n=10444)<br><br>Age (years) Mean±SD<br>(Pre-cancer depression cohort) 77.4± 6.9<br><br>Gender (Pre-cancer depression cohort)<br>Male (35%)<br><br>Ethnicity (Pre-cancer depression cohort)<br>White (90%); Hispanic (5%)	SEER-Medicare database, comprised of Medicare claims linked to clinical data for subjects in the National Cancer Institute's SEER dataset<br><br>Outcomes<br>Health care utilisation<br><br>Outcome measures<br>High intensity EoLC as chemotherapy in the last 14 days of life<br>Less than 3 days of hospice use or no hospice use<br>Any ICU admission last 30 days of life<br>>1 inpatient hospitalization admission last 30 days of life<br>In-hospital death<br>>1 ED visit in the last 30 days of life<br>Length of hospice enrolment
Study 9<br><br>Spilsbury et al 2018[38]<br>Australia<br><br>Aim<br>To describe the trajectory of acute care health service use in the LYoL for people with schizophrenia and how this varied with receipt of community-based SPC and morbidity burden<br><br>MH diagnosis<br>Schizophrenia<br><br>Cause of death (of relevance)<br>Heart failure; cirrhosis/liver disease, renal disease/dialysis, chronic lower respiratory disease or cancer<br><br>Definition of EoL/ PC<br>LYoL	Setting<br>Acute health care services and community-based health care services use within Western Australia<br><br>Participants<br>Decedents with a lifetime history of schizophrenia diagnoses (n=1196)<br>Matched sample of decedents with no previous schizophrenia diagnoses (n=62312)<br><br>Age (years) Mean±SD<br>(schizophrenia cohort)<br>20-29 years (4.3%); 30-39 years (7.4%)<br>30-39 years (10.9%); 50-59 years (14.2%)<br>60-69 years (17.7%); 70-79 years (19.1%)<br>80-89% (20.2%); 90+ years (6.4%)<br><br>Gender (schizophrenia cohort)<br>Male (52%)<br><br>Ethnicity<br>Not reported	Design<br>Retrospective Cohort (over 5 years)<br><br>Data sources<br>Data linkage and de-identified data extraction from the Western Australia Data Linkage System was performed<br><br>Outcomes<br>Acute care health service use<br>Receipt of community-based SPC<br>Morbidity burden<br><br>Outcome measure<br>Morbidity burden<br>ED presentations over LYoL<br>Number of hospitals admission in LYoL<br>Community based care in the LYoL

Study 10<br><br>Kelley-Cook et al 2016[33]<br>USA<br><br><u>Aim</u><br>To evaluate the clinical impact of PC in the treatment of terminally ill Vietnam Veterans with a history of PTSD versus those without PTSD, as it pertains to medications for symptom control at the EoL<br><br><u>Diagnosis</u><br>PTSD plus a range of other SMI's or SMI relevant symptoms e.g. hallucinations<br><br><u>Terminal conditions (of relevance)</u><br>Cancer, heart disease, liver disease, pulmonary disease and renal disease<br><br><u>Definition of EoL/ PC</u><br>Use of medications at EoL<br>Time frame not specified	<u>Setting</u><br>One US tertiary veterans' hospital<br><br><u>Participants</u><br>Veterans with PTSD (n=39)<br>Veterans without PTSD (n=137)<br><br><u>Age (years)</u> Mean$\pm$SD<br>Mean age was 65.12<br><br><u>Gender</u><br>Male (99%)<br><br><u>Ethnicity</u><br>Not reported	<u>Design</u><br>Retrospective cohort study<br><br><u>Data sources</u><br>Electronic medical records of a veteran's hospital<br><br><u>Outcomes</u><br>Social support<br>PC team interventions<br>Family meeting interventions<br>Medications<br><br><u>Outcome measures</u><br>Differences in pharmacologic treatment between those with and without PTSD<br>Nonpharmacological PC interventions between those with and without PTSD
Study 11<br><br>Cai et al 2011[27]<br>USA<br><br><u>Aim</u><br>To test whether persons with SMI are as likely as other nursing home residents to make informed choices about treatments through medical ACP<br><br><u>MH diagnosis</u><br>Schizophrenia, bipolar disorder, or other psychosis)<br><br><u>Terminal condition</u>	<u>Setting</u><br>Nursing homes (n=1,174)<br><br><u>Participants</u><br>Residents with SMI (n=1769) and those with no SMI (n=11738)<br><br><u>Age (years)</u> Mean$\pm$SD<br>SMI: 75.7$\pm$5<br>No SMI 81.1$\pm$2<br><br><u>Gender</u><br>SMI: Male (32%)<br>No SMI: Male (28.4%)<br><br><u>Ethnicity (</u>SMI)	<u>Design</u><br>Retrospective cohort study<br><br><u>Data sources</u><br>Data came from the 2004 National Nursing Home Survey that was conducted by the U.S. Centers for Disease Control and Prevention<br><br><u>Outcomes</u><br>Whether sampled residents had any of the following advance care documents collected by the survey: living wills, DNR orders, do-not hospitalize orders, and orders restricting feeding, medication, or other treatments

Not linked to a specific terminal condition<br><br><u>Definition of EoL/ PC</u><br>Medical ACP	non-Hispanic white (80.2%)<br>African American (13.3%)<br>Hispanic (5.3%), Other (1.2%)	
Study 12<br><br>Podymow et al 2006[37]<br>Canada<br><br><u>Aim</u><br>To improve health care delivery to homeless adults<br><br><u>MH diagnosis</u><br>Depression (43%)<br>Schizophrenia (39%)<br>Anxiety (18%)<br>None (14%)<br><br><u>Causes of death (of relevance)</u><br>Cancer (25%)<br>Chronic obstructive lung disease (4%)<br><br><u>Definition of EoL/ PC</u><br>PC delivery<br>Time frame not specified	<u>Setting</u><br>15-bed shelter-based PC/hospice pilot program<br><br><u>Participants</u><br>Homeless patients who were admitted to the Hospice, received terminal care and died between July 2001 and August 2003 (n=28)<br><br><u>Age (years)</u> Mean±SD<br>49± 10.5<br><br><u>Gender</u><br>Male (89%)<br><br><u>Ethnicity</u><br>Caucasian (89%); African American (7%)<br>Aboriginal (4%)	<u>Design</u><br>Retrospective cohort (over 2 years)<br><br><u>Data sources</u><br>Data obtained from physician and nurse's patient intake history and medical transfer notes<br><br><u>Outcomes</u><br>Clinical course and care of the patients<br>Costs<br><br><u>Outcome measures</u><br>Costs per patient stay<br>Main symptoms<br>Oxygen requirements<br>Pain management and type of pain management<br>PC consults<br>Number of medications<br>Type of self-care assistance<br>Religious counsel<br>Number of family visits<br>Reasons precluding transfer to PC hospital

Key: ACP: advance care plans; AD: advance directive: ED: emergency department; EoL: end of life; EoLC; end of life care; GP: general practitioners; ICU: intensive care unit; PTSD: post-traumatic stress disorder; SD: standard deviation; SEER: surveillance, epidemiology, and end results; SMI: severe mental illness; SPMI: severe and persistent mental illness

Characteristics of included qualitative studies

**Author, Year**<br>**Country**<br><br>**Aim**<br>**Mental health diagnosis**<br>**Terminal / Chronic condition or cause of death**<br>**Definition of EoL/ Palliative care**	**Setting**<br>**Patient characteristics**	**Design**<br>**Data collection methods**<br>**Data analysis**	**Thematic findings**
Study 13<br><br>Jerwood et al 2018[52]<br>UK<br><br>Aims:<br>To examine the views of the clinical staff on the barriers to providing EoLC to people with SMI and that begins to consider how improvements can be made<br><br>MH diagnosis<br>SMI but not linked to specific patients<br><br>Terminal condition<br>Not linked to a specific terminal condition<br><br>Definition of EoL/ PC<br>General EoLC	Setting<br>MH trust<br>Hospice<br><br>Participant<br>Clinical staff (n=23) who had some experience of working with the patient group and of the issues of EoLC<br><br>No patient details reported	Design<br>Qualitative descriptive<br><br>Data collection methods<br>Focus groups(n=4)<br><br>Cube ideas generation tool - Participants wrote and drew on the Cube while the verbal discussion was taking place. in addition, paper and envelopes were also made available to gather additional data from participants who felt uncomfortable sharing views in a group setting<br><br>Data analysis<br>Framework analysis	The structure of the system<br>The presentation of the patient<br>The confidence of the clinician<br>The problem of partnership
Study 14<br><br>McGrath and Holewa 2004[47]<br>McGrath & Jarrett 2007[48]<br>McGrath & Forrester 2006[49]<br>Australia<br><br>Aim of research project	Setting<br>MH institution<br><br>Participants<br>Those directly involved in the care of two patients who had died in the last year (n=8)	Design<br>Qualitative phenomenology<br><br>Data collection methods<br>Interviews<br><br>Participants were encouraged to tell their experience with caring for a terminally ill	McGrath and Holewa 2004[47]<br>Knowledge of PC<br>Definitions -educated guesses<br>Similarity to MH philosophy and practice<br>-A person-centred practice<br>-Relationship based connectedness<br>-Compassionate and Holistic Care

To document the experience of providing EoLC to patients for health care workers in an institutional MH setting.<br><br><u>Focus of individual publications</u><br>To highlight the similarity in philosophy between PC and MH practice[47]<br><br>The findings presented here focus on one of the central problems impacting on EoLC that of the stigma attached to MH patients[48]<br><br>To explore important ethico-legal issues at the interface of PC and institutional MH[49]<br><br><u>MH diagnosis</u><br>MH patients but not linked to specific patients<br><br><u>Terminal condition</u><br>Not linked to a specific terminal condition<br><br><u>Definition of EoL/ PC</u><br>General EoLC	No patient details reported	patient with the initial question of. Of particular interest was an exploration of factors that the participant believed either facilitated or hindered the provision of optimal EoLC<br><br><u>Data analysis</u><br>Thematic analysis	-Respect for autonomy and choice<br>-Concern for quality of life as defined by the client<br>-Focus on family as unit of care<br>-Multi-disciplinary teamwork<br>-Ability to face and deal with dying<br>-Death with dignity<br>-Concern to keep patient in familiar environment<br>-Special attribute of staff<br><br>McGrath & Jarrett 2007[48]<br>The MH stigma<br>-Suggested causes for the mental illness stigma<br>-Implications of mental illness stigma for EoLC<br>-Trained HCPs accompanying patient to PC facility<br>-The limited nature of positive assistance<br>-MH staff stress in caring for dying patients<br><br>McGrath & Forrester 2006<br>The legal dimension of care<br>-The coronial inquest<br>-Resuscitation versus being allowed to die
Study 15<br><br>McNamara et al 2018[51]<br>Australia<br><br><u>Aim</u>	<u>Setting</u><br>HCPs employed in government policy and management, PC, MH, community service provision, clinical care, allied health care, high care residential homes and community-based	<u>Design</u><br>Qualitative descriptive<br><br><u>Data collection methods</u><br>Interviews<br>Three participants who worked in the same location were interviewed together	People with schizophrenia at the EoL: challenges of a vulnerable group<br>- Individual factors that may affect people with schizophrenia at the EoL<br>- Social factors that may affect people with schizophrenia at the EoL

To address the paucity of research by documenting possible need, experiences of health care service use and factors affecting PC use for people with schizophrenia who have advanced life limiting illness<br><br><u>MH diagnosis</u><br>Schizophrenia but not linked to specific patients<br><br><u>Terminal condition</u><br>Advanced life limiting illness<br><br><u>Definition of EoL/ PC</u><br>General experiences EoLC	supported accommodation, including psychiatric hostels<br><br><u>Participants</u><br>HCPs (n=16) who were caring for, or had experience of caring for, people with schizophrenia<br>Three participants worked specifically in a role which covered both MH and PC<br><br>No patient details reported	An interview guide was used which focussed on the EoL needs of people with schizophrenia, their experiences with health services and their possible need for PC<br><br><u>Data analysis</u><br>Thematic analysis	- Health care factors that may affect people with schizophrenia at the EoL<br>- The interrelationship between factors that affect people with schizophrenia at the EoL<br><br>Barriers and facilitators to people with schizophrenia receiving PC<br>- Recognising declining health, communication and planning<br>- Collaboration and capacity building in the broader health, MH and PC sectors
Study 16<br><br>Morgan 2016[57]<br>USA<br><br><u>Aim</u><br>To explore the needs and attitudes of nurses in psychiatric/MH settings towards patients who are in need of palliative and end of- life care<br><br><u>MH diagnosis</u><br>SMI but not linked to specific patients<br><br><u>Terminal condition</u><br>Not linked to a specific terminal condition<br><br><u>Definition of EoL/ PC</u><br>General experiences EoL care	<u>Setting</u><br>Hospice / PC<br>Psychiatric / MH care<br><br><u>Participants</u><br>PC/HC nurses (n=7)<br>Psychiatric/MH Care (n=6)<br>PC/HC & MH Nurses (n=2)<br>HIV/AIDS nurses, geriatric nurse practitioner, and working with homeless individuals<br>All who had reported having worked with people with SMI at EoL<br><br>No patient details reported	<u>Design</u><br>Qualitative phenomenology describes as a pilot study<br><br><u>Data collection methods</u><br>Interviews<br><br>An interview guide was used to elicit the participants' stories related to the nurses' experience with EoL and PC with patients with SMI. The questions in the interview helped explore topics related to the specific aims and capture the impact of contextual and individual factors that influenced the individual's thought process, behaviour, and outcome<br><br><u>Data analysis</u><br>Thematic analysis	Stigma of mental illness<br>Effect of SMI symptoms on communication and trust<br>Chaotic family systems<br>Advocacy issues around pain, comfort care<br>Need for formal support and education for nurses<br>No right place to die
Study 17<br><br>Hackett & Gaitan 2007[55]	<u>Setting</u><br>Hospice	<u>Design</u><br>Qualitative grounded theory	How is MH assessed in the hospices?

UK<br><br><u>Aim</u><br>To gain an understanding of the current practice of MH assessments within the hospice service in two hospices in the UK<br><br><u>MH diagnosis</u><br>SMI but not linked to specific patients<br><br><u>Terminal condition</u><br>Not linked to a specific terminal condition<br><br><u>Definition of EoL/ PC</u><br>General experiences EoLC	<u>Participants</u><br>Nurses (n=6), HCAs (n=1) or doctors (n=2) recruited from two hospice staff teams<br><br>No further details reported	<u>Data collection methods</u><br>Interviews<br><br>An interview guide was used which focused on staff members' views on roles with regard to MH assessments and the overall processes involved<br><br><u>Data analysis</u><br>Constructing networks of findings based on the interview questions (How, Staff members; feelings, inform clinical practice and training needs)	What are the staff members' feelings about doing these assessments?<br><br>How does assessment information inform clinical practice?<br><br>What are the perceived training needs of staff?
Study 18<br><br>McKellar et al 2016[50]<br>Australia<br><br><u>Aim</u><br>To explore how Old Age Psychiatrists (OAP) approach and experience working with patients at the EoL<br><br><u>MH diagnosis</u><br>SMI but not linked to specific patients<br><br><u>Terminal condition</u><br>Not linked to a specific terminal condition<br><br><u>Definition of EoL/ PC</u><br>General experiences EoLC	<u>Setting</u><br>Public MH services for older people but most also maintained some private practice<br><br><u>Participants</u><br>Old age psychiatrists recruited via email sent to all members of Australian Faculty of Psychiatry of Old Age and New Zealand College of Psychiatrists (n=9)<br><br>No patient details reported	<u>Design</u><br>Qualitative descriptive<br><br><u>Data collection methods</u><br>Interviews<br><br>An interview guide, developed through literature review, provided a semi-structured approach, while allowing participants to freely develop topics. The literature review identified the variety of possible roles OAPs may have at the EoL, and these were used to formulate open-ended questions exploring participants' experiences working with patients at the EoL, approaches to this work and the impact of these experiences on participants<br><br><u>Data analysis</u><br>Thematic analysis	Death is not our business: working in a MH framework<br>- Death should not occur in psychiatry<br>- Working in a psychiatric treatment mode<br>- Keeping a distance from death<br>- Unexpected death is a negative experience<br>Death is our business: working in an aged care context<br>- Death is part of life<br>- Encountering the EoL through dementia care<br>- Doing EoL work<br>- Expected death is a positive experience

Study 19<br><br>Stajduhar et al 2019[56]<br>Canada<br><br><u>Aim</u><br>To identify barriers to accessing care among structurally vulnerable people at EoL<br><br><u>MH diagnosis</u><br>MH issues<br><br><u>Terminal condition</u><br>Not linked to a specific terminal condition<br><br><u>Definition of EoL/ PC</u><br>General experiences of accessing EoLC	<u>Setting</u><br>Homeless or vulnerably housed in a Western Canadian Province<br><br><u>Participants</u><br>Three participant groups<br>(1) those experiencing structural vulnerability and who were deemed to be on a palliative trajectory (n=25)<br>(2) their support (n=25)<br>(3) their formal service providers (housing workers, clinicians) (n=69)<br><br><u>Age (years)</u> (group 1)<br>Mean 59, range 19-81<br><br><u>Gender</u> (group 1)<br>Males (64%)<br><br><u>Ethnicity</u> (group 1)<br>White (52%); Indigenous (32%)<br>African Canadian (4%)	<u>Design</u><br>Qualitative ethnographic<br><br><u>Data collection methods</u><br>Ethnographical observation and in-depth interviews informed by the critical theoretical perspective of equity and social justice<br>Data collection for all groups was longitudinal, over 30 months via repeated observation in homes, shelters, transitional housing units, community-based service centres, on the street, and at health care appointments.<br><br>Semi-structured interviews and observational data Interviews (n=19 structurally vulnerable participants; n=16 support persons; n=23 service providers)<br><br><u>Data analysis</u><br>Constant comparison analysis<br>Thematic analysis	Barriers to accessing care<br>- Survival imperative<br>- Normalization of dying<br>- The problem of identification<br>- Professional risk and safety management<br>- Cracks of a 'silo-ed' care system
Study 20<br><br>Sweers et al 2013[54]<br>Belgium<br><br><u>Aim</u><br>To gain a better insight into the perspectives and expectations of patients with schizophrenia about EoL(care)<br><br><u>MH diagnosis</u><br>Schizophrenia<br>- Paranoid (65%)<br>- Schizoaffective disorder (35%)	<u>Setting</u><br>Psychiatric Hospital<br><br><u>Participants</u><br>Schizophrenic patients in remission with a minimum illness duration of 10 years (n=20)<br><br><u>Gender</u><br>Male (60%)<br><br><u>Age</u> (years)<br>Range 38-51 / Mean 47.3+6.9	<u>Design</u><br>Qualitative grounded theory<br><br><u>Data collection methods</u><br>Interviews<br>An interview guide was used and questions were broad and related to the experience of living with schizophrenia and EoL situations, associated feelings and reactions as well as the perceived impact of these experiences on the person's life and expectations about EoL(care)	Note Study conducted in Belgium where Euthanasia is legal.<br><br>Patients' EoL perspectives and expectations<br>- Fear of death<br>- Skilled companionship<br>- Quality of life<br>○ Physical well being<br>○ Psychological well being<br>○ Social concerns<br>○ Spiritual well being

 Terminal condition<br>Not linked to a specific terminal condition<br><br>Definition of EoL/ PC<br>Future EoL perspectives and expectations	Ethnicity<br>Not reported	5/10 interviewed twice - project refined due to lack of detailed information next 10 had 2 interviews each.<br><br>Data Analysis<br>Constant comparison analysis	
Study 21<br><br>Shulman et al 2018[53]<br>UK<br><br>Aim<br>To explore the views and experiences of current and formerly homeless people, frontline homelessness staff (from hostels, day centres and outreach teams) and health- and social-care providers, regarding challenges to supporting homeless people with advanced ill health, and to make suggestions for improving care<br><br>MH diagnosis<br>Background talks about tri-morbidity drug and/or alcohol dependence & MH problems in association with physical health issues<br><br>Terminal condition<br>Advanced life limiting illness<br><br>Definition of EoL/ PC<br>General experiences EoLC	Setting<br>Three London Boroughs<br><br>Participants<br>Single homeless people (n=28)<br>Formerly homes people (n=10)<br>Health and Social care providers (n=48)<br>Hostel staff (n=30)<br>Outreach staff (n=10)<br><br>Gender:<br>Male (54%) across all groups<br><br>No further patient details reported	Design<br>Qualitative descriptive<br><br>Data collection methods<br>Interviews (n=10)<br>Focus groups (n=28)<br><br>Use of case study vignette to generate discussion for all participants (no MH focus)<br>No details given re interviews<br><br>Data analysis<br>Thematic analysis	Complex behaviours in mainstream services<br>- Behaviours related to complex trauma and substance misuse issues; inflexibility and inexperience<br>Gaps in existing systems<br>- Lack of appropriate alternatives<br>- Need for holistic approach to care and support<br>- Hostel as a place of care and death<br>Uncertainty and complexity<br>- Difficulty predicting illness trajectories<br>- Advance care planning

Key: AIDS: acquired autoimmune deficiency syndrome; EoL: end of life; EoLC: end of life care; HC: hospice care; HCA: health care assistant; HCP: health care professionals; HIV: human immunodeficiency virus; MH: mental health; palliative care: PC; SMI: severe mental illness

Characteristics of included descriptive cross-sectional surveys

**Author, Year**<br>**Country**<br><br>**Aim**<br>**Mental health diagnosis**<br>**Terminal / Chronic condition or cause of death**<br>**Definition of EoL/ Palliative care**	**Setting**<br>**Place of death**<br>**Patient characteristics**	**Design**<br>**Data collection measures**<br>**Outcomes**
Study 22<br><br>Alici et al 2010[39]<br>USA<br><br><u>Aim</u><br>To define the frequency of PTSD-related symptoms among veterans who are near the EoL and to describe the impact that these symptoms have on patients and their families<br><br><u>MH diagnosis</u><br>PTSD related symptoms (17%)<br><br><u>Terminal condition</u><br>Not linked to a specific terminal condition<br><br><u>Definition of EoL/ PC</u><br>Last month of life	<u>Setting</u><br>VA medical centres and their affiliated nursing homes (n=5)<br><br><u>Participants</u><br>Family members of patients (n=524, rr 54%) who had received any inpatient or outpatient care from a participating VA medical centre in the last month of life. Of these were patients with PTSD-Related Symptoms (n =89)<br><br><u>Age (years)</u><br>Mean 71 (Range 50-94)<br><br><u>Gender</u><br>Male (99%)<br><br><u>Ethnicity</u><br>African American (20%)<br>White, non-Hispanic (78%)<br>Hispanic (1%); Other / unknown (1%)	<u>Design</u><br>Descriptive survey<br><br><u>Data collection methods</u><br>Questionnaire which assessed their perceptions of the quality of the care and services that the patients and they themselves received during the patients' last month of life and after the patients' deaths administered 4-6 weeks post death<br><br>The Family Assessment of Treatment at EoL (FATE) survey The instrument has 32 items that reflect key areas of PC outlined by current national guidelines. All items evaluated outcomes by asking respondents either for frequency ratings (e.g., ''How often did [the patient's] pain make him/her uncomfortable?'') or yes/no responses (e.g., ''Do you think [the patient] died where he/she wanted to?''). For each symptom that family members reported, they were asked how often that symptom made the patient uncomfortable (Never-0; Sometimes-1; Usually-2; Always-3)<br><br><u>Outcomes</u><br>Post-traumatic stress related symptoms<br>Post-traumatic stress disorder-related symptoms and satisfaction with care
Study 23<br><br>Patterson et al 2014[43]	<u>Setting</u><br>Inpatient and outpatient PC consult services within academic medical centre (non-profit,	<u>Design</u><br>Descriptive survey

USA<br><br><u>Aim</u><br>To explore the current level of collaboration between psychiatrists and PC consult services across the U.S. and the factors that support or restrict such involvement<br><br><u>MH diagnosis</u><br>Personality disorders (as reported by PC services) as well as other MH needs<br><br><u>Terminal condition</u><br>Not linked to a specific terminal condition<br><br><u>Definition of EoL/ PC</u><br>Use of PC services	community hospitals private medical centres and VA hospitals)<br><br><u>Participants</u><br>Program directors of PC consult services identified through the National Registry maintained by the Centre to Advance PC (n=260, rr =67%)<br><br>No patient details reported	<u>Data collection methods</u><br>A 36-item survey was developed based on consultation with a group of clinical and academic experts including two PC directors at academic medical centres, a PC psychiatrist, and a clinical psychologist. The survey was divided into four content areas: 1) demographic information about the program director, 2) information about the medical facility and composition of the PC service, 3) patient MH issues of concern to the PC service, and 4) psychiatrist involvement with the PC service.<br><br><u>Outcomes</u><br>Identified MH needs of PC patients<br>How comfortable the program directors reported being with treating the MH needs<br>Psychiatry involvement with PC services<br>Satisfaction with and desire for psychiatry involvement<br>Impediments to psychiatry involvement on PC services
Study 24<br><br>Evenblij et al 2016[58]<br>The Netherlands<br><br><u>Aim</u><br>To explore nurses' experiences with and to identify barriers to providing PC to psychiatric patients in Dutch MH facilities<br><br><u>MH diagnosis</u><br>Psychiatric disorders which included<br>Schizophrenic or other psychotic disorder (40%)<br>Personality or behavioural disorder (36%)<br>Mood disorder (30%)<br>Anxiety disorder (30%)<br><br><u>Causes of death (of relevance)</u><br>Cancer (35%);<br>Respiratory disorder (COPD) (4%)	<u>Setting</u><br>MH facilities<br><br><u>Participants</u><br>Nurses registered in the Dutch nurses' Association division of psychiatric nursing who currently worked in a MH facility and who had experience concerning PC provision (n=137, rr 26%)<br><br>No patient details reported	<u>Design</u><br>Mixed methods (descriptive survey and descriptive qualitative)<br><br><u>Data collection methods</u><br>An online survey based on pre-existing questionnaires. Pilot tested and minor changes made. Final survey consisted of two parts<br>All nurses were asked to fill in questions about their knowledge, experience and needs concerning PC provision (n = 137) and (2) nurses who had provided PC in the past 2 years were asked to fill in 27 additional case specific questions about the care they had provided to the last patient who died as a result of chronic physical comorbidity or old age (n = 50)<br>Only the case specific questions were reported in this publication<br><br>Face to face interviews (n=9) were conducted with a subset of the sample which sought to identify nurses' personal

Liver failure (4%)<br><br><u>Definition of EoL/ PC</u><br>General experience of PC services		experiences with PC provision in MH facilities. Thematic analysis was conducted.<br><br><u>Outcomes</u><br>Elements of PC provided (survey)<br>Thematic areas (interviews) - Barriers for PC provision / Physical care domain / Psychosocial care domain / Organisation
Study 25<br><br>Sheridan et al 2018[59]<br>Ireland<br><br><u>Aim</u><br>To examine how and by whom the need for PC is identified for people with a diagnosed mental illness – GPs, Psychiatrists, other HCPs<br>To identify the referral pathways and access to physical health and PC services<br>To identify how PC needs of people with SMI are managed once identified<br><br><u>MH diagnosis</u><br>SMI but not linked to specific patients<br><br><u>Terminal condition</u><br>Not linked to a specific terminal condition<br><br><u>Definition of EoL/ PC</u><br>General use of PC services	<u>Setting</u><br>General practice<br>Psychiatric practice<br><br><u>Participants</u><br>GPs who were currently on the register of the Irish College of General Practitioners in both Republic of Ireland (n=434, rr 17.4%) and Northern Ireland (n=133, rr 9%)<br>Psychiatrists (n=80 rr 26.2%) identified from the Irish Medical Directory<br><br>No patient details reported	<u>Design</u><br>Mixed methods (descriptive survey and qualitative descriptive)<br><br><u>Data collection methods</u><br>A postal survey was conducted, and the questionnaire was based on a similar questionnaire utilised with GPs in the Republic of Ireland, which was adapted and piloted. interviews with a subset of psychiatrists (n=6). No GPs and their existing patients with SMI in receipt of PC (n=2) agreed to take part.<br><br><u>Outcomes</u><br>Engagement with PC services (survey)<br>Opinions on needs and services (survey)<br>PC needs (survey)<br>Thematic areas (interviews)<br>Psychiatry –not a unitary practice<br>Understanding and managing PC<br>Accessing and negotiating the system;<br>Segregation, discrimination and stigma<br>Place of residence
Study 26<br><br>Evenblij et al 2019[46]<br>The Netherlands<br><br><u>Aim</u><br>To explore differences between care staff in MH facilities, nursing homes, and care	<u>Setting</u><br>MH facilities (Survey 1)<br>Nursing and care homes (Survey 2)<br><br><u>Participants</u><br>Nurses and care assistants who worked in MH facilities (n=137, rr 29%), nursing and care homes (n=440, rr 52%)	<u>Design</u><br>Descriptive survey<br><br><u>Data collection methods</u><br>Two descriptive surveys<br>Survey 1 see Evenblij et al 2016<br>Survey 2

homes with regard to knowledge about PC, time pressure, and self-efficacy in EoL communication, as well as aiming to identify determinants of high self-efficacy in EoL communication<br><br><u>MH diagnosis</u><br>SMI but not linked to specific patients<br><br><u>Terminal condition</u><br>Not linked to a specific terminal condition<br><br><u>Definition of EoL/ PC</u><br>EoL knowledge, self efficacy & communication	Patient characteristics not reported	Knowledge of PC was measured using the MOVE2PC questionnaire Assessing the time available for giving care to patients on an existing a 5 point scale tool developed by Ruijeters and Stevens<br>Self-efficacy in EoL communication was measured with the communication subscale from the S-EoLC survey.<br><br><u>Outcomes</u><br>Knowledge of the definition of PC<br>Time Pressures<br>Confidence in Staff's ability to engage in EoL communication<br>Determinants of high self-efficacy in EoL Communication
Study 27<br><br>Taylor et al 2012[44]<br>Australia<br><br><u>Aim</u><br>To improve the quality of PC for people with a severe and persistent mental illness and those who develop MH issues as a response to their diagnosis of a life-limiting illness<br><br><u>MH diagnosis</u><br>SMI but not linked to specific patients<br><br><u>Terminal condition</u><br>Not linked to a specific terminal condition<br><br><u>Definition of EoL/ PC</u><br>Training in PC for MH nurses	<u>Setting</u><br>A collaboration between one metropolitan PC service, the district nursing service, a MH service, and two regional PC services<br><br><u>Participants</u><br>MH workers (n=68), including consultant psychiatrists, GPs, MH nurses, RNs, ENs, social workers, OTs, counsellors, community outreach workers, paramedical assistants, and students of psychology and social work took part in PC workshops<br><br>PC professionals (n=36), including RNs, ENs, social workers, OTs, a bereavement counsellor, and an art therapist took part in MH workshops<br><br>Patient characteristics not reported	<u>Design</u><br>Descriptive case study with post- test evaluation<br><br><u>Intervention</u><br>Cross – training MH workshops for PC workers and PC workshops given to MH workers<br>Pre- and post-evaluations were conducted by an independent evaluator<br><br><u>Data collection methods</u><br>Self-assessment survey with open ended questions<br><br><u>Outcomes</u><br>Participants' knowledge before and after the workshop<br>The areas of learning considered to be of most importance
Study 28<br><br>Foti 2003[40]	<u>Setting</u><br>Two state hospitals and one acute community MH centre.	<u>Design</u>

USA<br><br><u>Aim</u><br>To better understand ACP processes for those with SMIs<br>To explore EoLC preferences amongst those with SMIs<br><br><u>MH diagnosis</u><br>SMI but not linked to specific patients<br><br><u>Terminal condition or cause of death</u><br>Not linked to a specific terminal condition<br><br><u>Definition of EoL/ PC</u><br>ACP	<u>Participants</u><br>Hospital patient records (n=328) and community MH inpatient records (n=16)<br><br>MH providers (nurse managers (n=4), nurses (n=7), case managers (n=14), community provider staff members (n=10), and supervisors (n=6)<br><br>Hospice and pastoral care providers (clinical staff (n=15), hospice directors (n=3), pastoral care providers (n=3)<br><br>*EoLC preferences*<br>People with SMI (no participants details reported)<br><br>*Education and training*<br>HCPs (no participants details reported)<br><br>*Professional outreach*<br>Medical professionals, MH advocacy groups and family and consumer groups (no participants details reported)<br><br>No further details reported	Mixed methods (descriptive survey consisting of a retrospective review of patients' medical records and a post-test evaluation of educational/training initiatives with HCPs<br><br><u>Intervention</u><br>The "Do It your Way project" involved education, training and outreach.<br>The educational and training initiatives involved cross training, neighbour to neighbour initiative and the development of education and training curricula. The outreach involved both professional outreach to medical professionals, MH advocacy groups, and family and consumer groups and patient outreach<br><br><u>Data collection methods for needs assessment</u><br>Medical records analysis of hospital patient records and community MH inpatient records and core competence and comfort assessment undertaking using a survey of HCPs<br><br><u>Data collection methods for the "Do It your Way project"</u><br>*EoLC preferences*<br>To explore EoLC preferences amongst those with SMIs the team developed the 21 item HCPQ. To specifically evaluate the subject's capacity to select a health care agent the team developed the CAT-HCP<br><br>*Education and training*<br>Post cross training core competency assessments<br><br>*Professional outreach*<br>Project staff developed a survey of attitudes and perceptions regarding ACP for persons with SMI.<br><br><u>Outcomes for needs assessment</u><br>Evidence of actually having completed a proxy designation<br>Formal education as well as personal and professional experiences regarding ACP and EoLC / Improvements for care

		<u>Outcomes for the "Do It your Way project"</u><br>Number of people receiving assistance with ACP<br>Project impact in the form of preliminary findings<br>Comfort and confidence in skills after cross training
Study 29<br><br>Foti et al 2005a/b[41,41]<br>USA<br><br><u>Aim</u><br>To ascertain preferences for EoLC among persons with serious mental illness<br>To examine preferences regarding medical ACP among persons with serious mental illness, specifically, experience, beliefs, values, and concerns about health care proxies and EoL issues<br><br><u>MH diagnosis</u><br>Schizophrenia or other psychosis (66%)<br>Mood disorders (major depression and bipolar disorder) (28%)<br>Personality disorders or PTSD (6%)<br><br><u>Terminal condition</u><br>Not linked to a specific terminal condition<br><br><u>Definition of EoL/ PC</u><br>Preferences for EoLC; ACP	<u>Setting</u><br>Five community MH agencies. Two agencies were located in suburban settings and three in urban environments.<br><br><u>Participants</u><br>Adults with 'serious mental illness who were receiving community-based services (n=150)<br><br><u>Age (years)</u> Mean+SD<br>42±10<br><br><u>Gender</u><br>Male (55%)<br><br><u>Ethnicity</u><br>Caucasian (91%)<br>Other (9%)	<u>Design</u><br>Descriptive survey<br><br><u>Data collection methods</u><br>HCPQ (see Foti et al 2003)<br>Two hypothetical health state scenarios were adapted from standard scenarios in which patients have conditions that prevent them from expressing a choice between different types of PC, aggressive treatments, and life support. The authors anticipated that hypothetical health state scenarios would enable us to learn about the ACP preferences of persons with serious mental illness, as they have for the general population<br><br><u>Outcomes</u><br>EoLC preferences under the scenario of terrible pain from terminal metastatic cancer<br>Preferences for life support in the event of brain damage with little chance of recovery, both for an imaginary other patient and for the respondent (self), among persons with SMI experience with ACP<br>Beliefs, values and EoL concerns<br>Characteristics associated with EoL preferences
Study 30<br><br>Elie et al 2018[45]<br><br>Canada<br><br><br><u>Aim</u>	<u>Setting</u><br><br>Jewish General Hospital<br><br><br><u>Participants</u>	<u>Design</u><br><br>Descriptive survey<br><br><br><u>Data collection methods</u><br><br>The authors conducted a 20-minute semi-structured interview based on the HCPQ to gather information on different end-of

To compare SPMI an CMI patients' EoLC preferences and comfort level with EoLC discussion<br><br>To identify potential predictors of interest in requesting medical assistance in dying at the EoL<br><br><u>MH diagnosis</u><br><br>SPMI (53%)<br><br><u>Terminal condition</u><br><br>Not linked to a specific terminal condition for those with SPMI. However, 47% were CMI<br><br><u>Definition of EoL/ PC</u><br><br>Preferences and comfort level with EoLC discussion<br><br>Medical assistance in dying	Patients attending the hospital, between May and July 2016.<br><br>Study group: patients diagnosed with a SPMI by their treating psychiatrist and without severe behavioral disturbances (n=106, rr 83%)<br><br>Comparator group: patients diagnosed with at least one chronic medical illness for over 2 years (n=95, rr89%)<br><br><u>Age (years) </u>Mean+SD<br><br>SPMI: 65.5+12.6; CMI: 63.3+12.6<br><br><u>Gender</u><br><br>SPMI: Male (33%); CMI: Male (43%)<br><br><u>Ethnicity</u><br><br>SPMI: Caucasian (80%), non-Caucasian (20%)<br><br>CMI: Caucasian (58%), non-Caucasian (42%)	life care topics such as treatment preferences and comfort level about EoL discussion. Two imaginary scenarios were also explored with participants (see Foti et al 2005aq/b).<br><br><u>Outcomes</u><br><br>Patients' agreement to use pain medication, palliative sedation, and MAID in case of terminal cancer<br><br>Preferred disposition regarding artificial life support in case of permanent brain damage

Key: ACP: advance care planning; CAT-HCP: Competency Assessment Tool-Health Care Proxy; CVA: cardio vascular accident, CMI: chronic medically ill; COPD: chronic obstructive airways disease; ED: emergency department; EoL: end of life; EoLC: end of life care; EN: enrolled nurse; GPs: general practitioners; HCP: health care professionals: HCPQ: Health Care Preferences Questionnaire; ICU: intensive care unit; MH: mental health; OT: occupational therapist; PTSD: post-traumatic stress disorder; rr: response rate; RN: registered nurse: SD: standard deviation; SEER: surveillance, epidemiology, and end results; SMI: severe mental illness; SPMI: severe and persistent mental illness; U.S.: United States; VA: veteran affairs
