# Supplementary material for: End of life care for people with severe mental illness: Mixed methods systematic review and thematic synthesis (the MENLOC study)
Source: Palliat Med. 2021 Sep 3;35(10):1747–60. doi: 10.1177/02692163211037480 (PMC8637363; doi:10.1177/02692163211037480)
Supplement: sj-pdf-3-pmj-10.1177_02692163211037480 – Supplemental material for End of life care for people with severe mental illness: Mixed methods systematic review and thematic synthesis (the MENLOC study) [file sj-pdf-3-pmj-10.1177_02692163211037480.pdf]

Critical appraisal scores for cohort studies

Citation	Q1	Q2	Q7	Q9	Q10	Q11	Q13	Q14	Overall Assessment
1. Butler and O'Brien 2018[26]	Y	Y	Y	Y	Y	Y	Y	N	Acceptable
2. Chochinov et al 2012[28] Martens et al 2013[35]	Y	Y	Y	Y	Y	Y	Y	Y	Acceptable
3. Ganzini et al 2010[30]	Y	Y	Y	Y	Y	Y	N	N	Acceptable
4. Huang et al 2017[31]	Y	Y	Y	Y	Y	Y	Y	N	Acceptable
5. Huang et al 2018[32]	Y	Y	Y	Y	Y	Y	Y	Y	Acceptable
6. Lavin et al 2017[34]	Y	Y	Y	Y	Y	Y	Y	N	Acceptable
7. McDermott et al 2018[36]	Y	Y	Y	Y	Y	Y	Y	Y	Acceptable
8. Podymow et al 2006[37]	Y	Y	Y	Y	Y	Y	N	N	Acceptable
9. Spilsbury et al 2018[38]	Y	Y	Y	Y	Y	Y	N	N	Acceptable
10. Fond et al 2019[29]	Y	Y	Y	Y	Y	Y	Y	Y	Acceptable
11. Cai et al 2011[27]	Y	Y	Y	Y	Y	Y	Y	Y	Acceptable
12. Kelley-Cook 2016[33]	Y	Y	Y	Y	Y	Y	Y	N	Acceptable

Key: Y=Yes, N=No

Critical appraisal scores for descriptive studies

Citation	Q1	Q2	Q3	Q4	Q5	Q6	Q7	Q8	Q9	Q10	Q11	Q12
1. Alici et al 2010[39]	Y	Y	U	Y	Y	Y	Y	Y	Y	Y	Y	Y
2. Elie et al 2018[45]	Y	Y	Y	Y	Y	Y	Y	Y	Y	Y	Y	Y
3. Evenblij et al 2016[58]	Y	Y	Y	Y	Y	Y	Y	Y	Y	Y	N	Y
4. Evenblij et al 2019[46]	Y	Y	Y	Y	Y	Y	Y	Y	Y	Y	Y	Y
5. Foti 2003[40]	Y	Y	Y	U	N	Y	N	N	N	N	N	N
6. Foti et al 2005[42] Foti et al 2005[41]	Y	Y	Y	Y	Y	Y	Y	Y	Y	Y	Y	Y
7. Patterson et al 2013[43]	Y	Y	Y	Y	Y	Y	Y	Y	Y	Y	Y	Y
8. Sheridan et al 2018[59]	Y	Y	Y	Y	Y	Y	Y	Y	Y	Y	N	Y
9. Taylor et al 2013[44]	Y	Y	N	N	U	N	N	N	N	N	Y	N

Key: Y=Yes, N=No, U=Unclear

Critical appraisal scores for qualitative studies

Citation	Q1	Q2	Q3	Q4	Q5	Q6	Q7	Q8	Q9	Q10
1. Evenblij et al 2016[58]	Y	Y	Y	Y	Y	N	Y	Y	Y	Y
2. Hackett and Gaitan 2007[55]	Y	Y	Y	Y	Y	Y	Y	Y	Y	Y
3. Jerwood 2018[52]	Y	Y	Y	Y	Y	Y	Y	Y	Y	Y
4. McGrath and Forrester 2006[49] McGrath and Holewa 2004[47] McGrath and Jarrett 2007[48]	Y	Y	Y	Y	Y	N	Y	Y	Y	Y
5. McKellar et al 2015[50]	Y	Y	Y	Y	Y	Y	Y	Y	Y	Y
6. McNamara et al 2018[51]	Y	Y	Y	UC	Y	N	Y	Y	Y	Y
7. Morgan et al 2016[57]	Y	Y	Y	Y	Y	N	Y	Y	Y	Y
8. Sheridan et al 2018[59]	Y	Y	Y	Y	Y	N	Y	Y	Y	Y
9. Shulman et al 2018[53]	Y	Y	Y	Y	Y	N	Y	Y	Y	Y
10. Stajduhar et al 2019[56]	Y	Y	Y	Y	Y	Y	Y	Y	Y	Y
11. Sweers et al 2013[54]	Y	Y	Y	Y	Y	N	Y	Y	Y	Y

Key: Y=Yes, N=No, U=Unclear
