# Supplementary material for: End of life care for people with severe mental illness: Mixed methods systematic review and thematic synthesis (the MENLOC study)
Source: Palliat Med. 2021 Sep 3;35(10):1747–60. doi: 10.1177/02692163211037480 (PMC8637363; doi:10.1177/02692163211037480)
Supplement: sj-pdf-4-pmj-10.1177_02692163211037480 – Supplemental material for End of life care for people with severe mental illness: Mixed methods systematic review and thematic synthesis (the MENLOC study) [file sj-pdf-4-pmj-10.1177_02692163211037480.pdf]

CERQual qualitative evidence profile, synthesis 1: Structure of the system

Review Finding<br><br>Studies Contributing to the Review Finding	Assessment of Methodological Limitations	Assessment of Relevance	Assessment of Coherence	Assessment of Adequacy	Overall CERQual Assessment of Confidence
**Structure of the system: Policy and guidance**					
1. Due to a lack of national and local guidance regarding EoLC and MH care, PC nurses report concerns about their legislative responsibility<br>Study 13	No methodological limitations	Serious concerns about relevance as only one study representing one country contributed to this finding	No concerns about coherence	Serious concerns about adequacy (only one study offering data)	Very low confidence
**Structure of the system: Separate commissioning, management and organisation**					
**Accessing and navigating the system**					
The separate commissioning, management and organisation of MH and EOL often result in those with SMI having trouble accessing and navigating the system[61–63]					Ungraded – non research
2.HCPs recognise that accessing and navigating EoLC for those with SMI is a challenge and many obstacles exist<br>Studies 14, 15, 25	Very minor methodological limitations (all studies had very minor methodological limitations)	Moderate concerns about relevance (partial relevance, as the studies were from two countries only which were Ireland and Australia)	Moderate concerns about coherence (data more varied and this finding is over-simplified)	Minor concerns about adequacy (three studies with moderately rich data overall)	Moderate confidence
**Access for homeless and vulnerable groups**					
Access to health care is particularly difficult for homeless and vulnerable groups, who are often isolated and disconnected from family and friends[64–66]					Ungraded – non research

3 Homeless people are often excluded from hospices and care homes, and HCPs report difficulties in placing homeless people with cancer<br>Studies 19, 21	No or very minor methodological limitations (one study with very minor and one study with no methodological limitations)	Moderate concerns about relevance (partial relevance, as the studies were from two countries only which were UK and Canada	Moderate concerns about coherence (data more varied and this finding is over-simplified)	Very minor concerns about adequacy (two studies with rich data)	Moderate confidence
**Care coordination across systems**					
It is important that care is coordinated and integrated across services so that the needs of those with SMI at EoL are met, although it is acknowledged that this is difficult to achieve[62,64,67,68]					Ungraded – non research
4. Separate commissioning, management and ways of organising both services and professionals create challenges for both MH and EoL HCPs to coordinate high quality care across MH and EoLC systems<br>Study 13	No methodological limitations	Serious concerns regarding relevance as only one study representing one country contributed to this finding	No concerns about coherence	Serious concerns about adequacy (only one study offering data)	Very low confidence
5. The invisibility of homeless and vulnerable people, and the fragmented character of care systems, means they often have to act as their own care coordinators<br>Studies 13, 19, 21	No or very minor methodological limitations (two studies with no and one study with very minor methodological limitations	Moderate concerns about relevance (partial relevance, as the studies were from only UK)	No concerns about coherence	Minor concerns about adequacy (two studies with rich data and one study with thin data)	Moderate confidence
6. Multiple proposals have been made to improve the coordination of care for people with SMI at the EoL, mostly involving the identification of staff with clear responsibilities	Minor methodological limitations (two studies with very	Moderate concerns about relevance (partial relevance, as the studies were from three	Minor concerns about coherence (data	Minor concerns about adequacy (two studies with rich data and	Moderate confidence

Studies 16, 25, 27	minor and one study with serious methodological limitations)	countries only which were Ireland, Australia and USA)	reasonably consistent within and across all studies)	one study with thin data)	
**Resources**					
7. HCPs feel that there is a lack of resources in terms of services and trained professionals that are able to meet the needs of those with SMI at EoL<br>Studies 23, 25, 28	Minor methodological limitations (one study with no, one study with very minor and one study with serious methodological limitations)	Moderate concerns about relevance (partial relevance, as the studies were from two countries only which were Ireland and Australia)	No concerns about coherence	Minor concerns about adequacy (two studies with rich data and one study with thin data)	Moderate confidence
**Structure of the system: Partnership**					
**Funding and flexibility to work in partnership**					
8. Working in partnership across EoL and MH services is important and when flexibility and solutions to work in partnership across agencies exist then EoLC for those with SMI can be facilitated<br>Study 28	No methodological limitations	Serious concerns regarding relevance as only one study representing one country contributed to this finding	No concerns about coherence	Serious concerns about adequacy (only one study offering data)	Very low confidence
9. A lack of good relationships and partnership working, influenced by reductions in funding and competitive commissioning, inhibit good care especially with regard to pain management, needs assessments and assessment of mental capacity<br>Study 13, 15, 25	Very minor methodological limitations (all studies had very minor methodological limitations)	Moderate concerns about relevance (partial relevance, as the studies were from three countries only which were Ireland, Australia and UK	No concerns about coherence	Minor concerns about adequacy (two studies with rich data and one study with thin data)	Moderate confidence
**Multidisciplinary teamwork**					

Interagency and interprofessional team working at EoL is important[64,68–72]					Ungraded – non research
10. HCPs report that formal (and sometimes ad-hoc) multidisciplinary teamwork can improve patient outcomes<br>Study 14, 15, 18, 28	Minor methodological limitations (one study with one with no, one study with very minor, one study with minor and one study with serious methodological limitations)	Moderate concerns about relevance (partial relevance, as the studies were from two countries only which were Australia and USA)	Moderate concerns about coherence (data more varied and this finding is over-simplified)	Minor concerns about adequacy (three studies with rich data and one study with thin data)	Moderate confidence
**Ongoing interprofessional communication**					
Ongoing communication between partners is a key element of high-quality care[62,64,70]					Ungraded – non research
11. Coalition-building and formal opportunities to meet and discuss care are essential and must ensure information is made available to primary care and social services partners<br>Studies 13, 15, 25, 28	Minor methodological limitations (one study with no, two studies with very minor methodological limitations and one study with serious methodological limitations)	Minor concerns about relevance (studies of HCPs representing a variety of settings and from across four countries which were Ireland, Australia, UK and USA, covering three continents)	Minor concerns about coherence (data reasonably consistent within and across all studies)	Minor concerns about adequacy (two studies with rich data and two studies with thin data)	High confidence
**Structure of the system: No right place to die**					
**Dying at home**					

With the right support from community based services, people with SMI at EoL are able to stay at home[61]					Ungraded – non research
12. MH and EoL staff recognise that allowing people to die in the location of their choice (which is often a home or home, like environment) is important, but staff also talk about how appropriate care is often lacking in all settings<br>Studies 15, 16, 25	Very minor methodological limitations (all studies had very minor methodological limitations)	Moderate concerns about relevance (partial relevance, as the studies were from three countries only which were Australia, Ireland and USA)	No concerns about coherence	No concerns	High confidence
**Dying in a mental health hospital**					
13. MH services rarely care for people with SMI at EOL as they are poorly equipped to meet their needs, and as a result patients are frequently moved between services<br>Studies 13, 16, 24	No or very minor methodological limitations (one study with no and two studies with very minor methodological limitations)	Moderate concerns about relevance (partial relevance, as the studies were from three countries only which were UK, USA and Netherlands)	No concerns about coherence	Minor concerns about adequacy (two studies with rich data and one study with thin data)	High confidence
**Dying in a hospice**					
14. Multiple challenges exist in order for EoLC to take place in a hostel, including lack of staff preparedness, the chaotic environment, concerns over risks and the safe storage of medication<br>Studies 12, 21	Very minor methodological limitations (all studies had very minor methodological limitations)	Moderate concerns about relevance (partial relevance, as the studies were from two countries only which were UK and Canada)	Moderate concerns about coherence (data more varied and this finding is over-simplified)	Very minor concerns about adequacy (two studies with rich data)	Moderate confidence
**Dying in acute settings**					
15. Providing EoLC in the community, within MH settings or homeless shelters can be difficult and as a result those with SMI are often transferred into acute settings at EoL but	No or very minor methodological limitations (three studies with no	Minor concerns about relevance (studies of HCPs representing a variety of settings and	Moderate concerns about coherence (data more	Minor concerns about adequacy (seven studies with rich data	Moderate confidence

providing MH care in acute settings also poses challenges<br>Studies 13, 15, 16, 18, 19, 24, 25	and five studies with very minor methodological limitations)	from across five countries which were Ireland, Australia, UK, USA and Netherlands across three continents	varied and this finding is over-simplified)	and one study with thin data)	
**Dying in hospice**					
16. Staff working within hospices report that hospices are ill-prepared for caring for the needs of homeless people at the EoL and require more help in dealing with substance misuse and the alternative lifestyles of is mostly a younger age group<br>Studies 19, 21	No or very minor methodological limitations (one study with no and one study with very minor methodological limitations)	Moderate concerns about relevance (partial relevance, as studies were from two countries only which were UK and Canada	Minor concerns about coherence (data reasonably consistent within and across all studies)	Very minor concerns about adequacy (two studies with rich data)	Moderate confidence

The synthesis findings presented here are drawn from the wider thematic synthesis undertaken for this review. The themes identified were summarised into evidence statements, as illustrated in this table

Key: EoL: End of Life; EoLC: End of Life Care; HCP: Health Care Professional; MH: Mental Health; PC: Palliative Care.

CERQual qualitative evidence profile, synthesis 2: Professional issues

**Review Finding**<br><br>**Studies Contributing to the Review Finding**	**Assessment of Methodological Limitations**	**Assessment of Relevance**	**Assessment of Coherence**	**Assessment of Adequacy**	**Overall CERQual Assessment of Confidence**
**Professional issues: Relationships between health care professionals and people with severe mental illnesses**					
**Connecting relationships**					
1. Some MH staff feel that building nurturing relationships are important especially for those with limited social networks and no family contact. However, others choose not to form such relationships finding it too upsetting when patients are transferred for EoLC<br>Studies 14-16, 24, 25	Very minor methodological limitations (all studies had very minor methodological limitations)	Minor concerns about relevance (studies of HCPs representing a variety of settings and from across four countries which were Ireland, Australia, Netherlands and USA, covering three continents)	Moderate concerns about coherence (data more varied and this finding is over-simplified)	No concerns	High confidence
**Talking about death and dying**					
People with life-limiting illnesses (including SMI) should be supported to make decisions at the EoL and good communication is important[62,73]					Ungraded – non research
2. Some MH staff find conversations about death and dying challenging those who feel able have found that patients are receptive<br>Studies 13, 15, 24, 26	No or very minor methodological limitations (two studies with very minor and one study with no methodological limitations)	Moderate concerns about relevance (partial relevance, as the studies were from three countries only which were Australia, Netherlands and UK)	No concerns about coherence	Minor concerns about adequacy (three studies with rich data and one study with thin data)	High confidence
**Attitudes and beliefs of health care professionals**					

3. The underlying stigmatising and prejudicial attitudes of EoL HCPs towards those with SMI including those who are homeless can affect decisions around EoLC<br>Studies 13, 14, 16, 17, 19, 21, 25	No or very minor methodological limitations (five studies with very minor and three studies with no methodological limitations)	No concerns	No concerns about coherence	Minor concerns about adequacy (seven studies with rich data and one study with thin data)	High confidence
**Professional issues: Mental health professionals doing end of life care**					
4. MH staff feel that they have limited experience of caring for patients with SMI at EOL and although some feel able to deliver care others feel that they lack the knowledge and skills, particularly with regard to pain management and psychosocial or spiritual support<br>Studies 13, 14, 24	No or very minor methodological limitations<br>(two studies with very minor and one study with no methodological limitations)	Moderate concerns about relevance (partial relevance, as the studies were from three countries only which were Australia, Netherlands and UK)	Minor concerns about coherence (data reasonably consistent within and across all studies)	Minor concerns about adequacy (two studies with rich data and one study with thin data)	Moderate confidence
**End of life care not being mental health work**					
5. Some MH staff report that they do not feel able, are not interested or avoid caring for people with SMI at EoL whereas others embrace caring for them<br>Studies 13, 16, 18, 23, 24	No or very minor methodological limitations<br>(two studies with very minor and three studies with no methodological limitations)	Minor concerns about relevance (studies of HCPs representing a variety of settings and from across four countries which were Netherlands, Australia, UK and USA, covering three continents)	Moderate concerns about coherence (data more varied and this finding is over-simplified)	Minor concerns about adequacy (four studies with rich data and one study with thin data)	Moderate confidence
**Emotional distress**					
6. Mental health staff report that caring for those with SMI at EOL can be distressing and	No or very minor methodological	Moderate concerns about relevance (partial	Moderate concerns	No concerns	Moderate confidence

emotionally draining, and some fear being scrutinised following the death of patients<br>Studies 14, 16, 17	limitations (two studies with very minor and one study with no methodological limitations)	relevance, as the studies were from three countries only which were Australia, USA and UK)	about coherence (data more varied and this finding is over-simplified)		
Staff who provide care over long periods to individuals with SMI need support in the face of terminal illness, and in bereavement[64]					Ungraded – non research
**Professional issues: End of life health care professionals doing mental health care**					
**Experience, knowledge and skills**					
7. End of life HCPs feel that they lack knowledge and understanding of MH diagnoses and services. As a result, they report that they are not always confident, willing or comfortable to care for patients with SMI at EoL<br>Studies 13-17, 23, 24	No or very minor methodological limitations<br>(four studies with very minor and three studies with no methodological limitations)	Minor concerns about relevance (studies of HCPs representing a variety of settings and from across four countries which were Netherlands Australia, UK and USA, covering three continents)	Moderate concerns about coherence (data more varied and this finding is over-simplified)	Minor concerns about adequacy (six studies with rich data and one study with thin data)	Moderate confidence
As a consequence of this, the needs of people with SMI at EoL are not always well met[64,74]					Ungraded – non research
End of life HCPs should become conversant with the needs of people with SMI and work closely with MH services[64]					Ungraded – non research
**Professional issues: Training and education**					
8. MH and EOL HCPs have highlighted a wide range of educational needs<br>Studies 14-17, 28	Minor methodological limitations (one study with no, three studies with very minor and	Moderate concerns about relevance (partial relevance, as the studies were from three countries	Moderate concerns about coherence (data more varied and this	Minor concerns about adequacy (three studies with rich data	Moderate confidence

	one study with serious methodological limitation)	only which were Australia, USA and UK)	finding is over-simplified)	and one study with thin data)	
Education and training opportunities in EoLC across professional groups who care for people with SMI at EoL are important[62,62,64,64,65,69,75–77]					Ungraded – non research
9. Palliative care program directors have suggested that psychiatry training is inadequate in the categories of EoLC and issues related to death and dying<br>Study 23	No minor methodological limitations	Serious concerns regarding relevance as only one study representing one country contributed to this finding	No concerns about coherence	Serious concerns about adequacy (only one study offering data)	Very low confidence
10. Whilst some MH nurses feel that that their core professional preparation enables them to care for people with SMI during periods of physical illness and at EoL others feel that this was something that could be improved<br>Studies 14, 16	Very minor methodological limitations (all studies had very minor methodological limitations)	Moderate concerns about relevance (partial relevance, as the studies were from two countries only which were Australia and USA)	Minor concerns about coherence (data reasonably consistent within and across all studies)	Very minor concerns about adequacy (two studies with rich data)	Moderate confidence
In-service training opportunities for HCPs to develop skills and knowledge in EoLC are patchy, particularly in mental health care-providing organisations and could be improved[62,64,65,72,76,78]					Ungraded – non research
End of life and MH staff working together can improve knowledge and awareness, such as through the establishment of dedicated link					Ungraded – non research

positions with roles including the provision of education[64]					
11. End of life and MH staff learning from each other such as through cross training has been positively evaluated<br>Studies 27, 28	Serious methodological limitations (two studies with serious methodological limitations)	Serious concerns regarding relevance as only two studies representing one country contributed to this finding	No concerns regarding coherence	Serious concerns about adequacy (two studies offering thin data)	Very low confidence

The synthesis findings presented here are drawn from the wider thematic synthesis undertaken for this review. The themes identified were summarised into evidence statements, as illustrated in this table

Key: EoL: End of Life; EoLC: End of Life Care; HCP: Health Care Professional; MH: Mental Health; SMI: Severe Mental Illness.

CERQual qualitative evidence profile, synthesis 3: Contexts of care

**Review Finding**<br><br>**Studies Contributing to the Review Finding**	**Assessment of Methodological Limitations**	**Assessment of Relevance**	**Assessment of Coherence**	**Assessment of Adequacy**	**Overall CERQual Assessment of Confidence**
**Contexts of care: Managing the interface between mental health and end of life care**					
**General practitioners managing care**					
1. General practitioners are the gatekeepers in enabling or hindering access to PC services and in some instances manage the PC needs of people with SMI within their practice<br>Studies 24, 25	Very minor methodological limitations (all studies had very minor methodological limitations)	Moderate concerns about relevance (partial relevance, as the studies were from two countries only which were Netherlands and Ireland)	Moderate concerns about coherence (data more varied and this finding is over-simplified)	Very minor concerns about adequacy (two studies with rich data)	Moderate confidence
**The role of medical specialists**					
2. People with SMI are reported to visit a number of different types of physician specialists as well as psychiatrists at EoL<br>Studies 23-25	No or very minor methodological limitations (one study with no and two studies with very minor methodological limitations)	Moderate concerns about relevance (partial relevance, as the studies were from three countries only which were USA, Netherlands and Ireland)	Minor concerns about coherence (data reasonably consistent within and across all studies)	No concerns about adequacy	Moderate confidence
**Referral**					
Support for people living with MH issues and approaching the EoL must be fast-tracked or prioritised[62]					Ungraded – non research

3. Both EoL and MH staff acknowledge that referrals are often complicated and lack vital information that would facilitate communication between themselves and those with SMI at EOL<br>Studies 13, 15	No or very minor methodological limitations (one study with no and one study with very minor methodological limitations)	Moderate concerns about relevance (partial relevance, as the studies were from two countries only which were Australia and UK)	Moderate concerns about coherence (data more varied and this finding is over-simplified)	Moderate concerns about adequacy (one study offering moderately rich data and one study offering thin data)	Low confidence
**Mental health assessment at end of life**					
Skilled MH assessment at EoL is required for people with SMI so that care can be planned and they can be helped to manage their symptoms and other needs[64]					Ungraded – non research
4. Both EoL and MH HCPs across a variety of settings report that, MH assessments at EoL can be a challenge and that they need help to deal with their fears and uncertainties<br>Studies 13, 17, 18, 25	No or very minor methodological limitations (three studies with no and two studies with very minor methodological limitations)	Moderate concerns about relevance (partial relevance, as the studies were from three countries only which were Australia and UK and Ireland)	Moderate concerns about coherence (data more varied and this finding is over-simplified)	Minor concerns about adequacy (four studies with rich data and one study with thin data)	Moderate confidence
**Health care services and treatment utilisation in the last year of life**					
**Palliative care**					
5. General practitioners and psychiatrists believe that people with SMI are less likely than other groups to make use of SPC<br>Study 25	No methodological limitations	Serious concerns about relevance as only one study representing one country contributed to this finding	No concerns about coherence	Serious concerns about adequacy (only one study offering data)	Very low confidence
**Invasive interventions**					
6. EoL HCPs working in the UK feel that standard guidance on resuscitation is lacking for people with SMI at EoL	No methodological limitations	Serious concerns about relevance as only one study representing one	No concerns about coherence	Serious concerns about adequacy (only	Very low confidence

Study 13		country contributed to this finding		one study offering data)High	
7. EoL professionals in Australia have concerns about capacity to consent in relation to resuscitation orders for people with SMI at EoL<br>Study 14	Very minor methodological limitations	Serious concerns about relevance as only one study representing one country contributed to this finding	No concerns about coherence	Serious concerns about adequacy (only one study offering data)	Very low confidence
**Contexts of care: Meeting individual and family needs**					
**Spiritual and psychosocial support**					
People with SMI have particular vulnerabilities arising from their MH experiences and programmes and services for people with SMI at EoL require a comprehensive team approach[62,64,73]					Ungraded – non research
8. Programmes and services for people with SMI at EoL require a comprehensive team approach incorporating symptom relief, psychological and psychosocial support and spiritual care<br>Studies 10, 12, 14, 15, 17, 24	No or very minor methodological limitations (one study with no and five studies with very minor methodological limitations)	Minor concerns about relevance (studies of HCPs and families representing a variety of settings and from across four countries which were Netherlands, Australia, UK and USA, covering three continents)	Moderate concerns about coherence (data more varied and this finding is over-simplified)	No concerns	High confidence
**Families and their involvement**					
9. HCPs report challenges in handling contact with families especially where years of estrangement have occurred or where family members also have MH conditions<br>Studies 12-16, 24	No or very minor methodological limitations (one study with no and five studies with very minor	Minor concerns about relevance (studies of HCPs representing a variety of settings and from across four countries which were Netherlands,	Moderate concerns about coherence (data more varied and this	Minor concerns about adequacy (five studies with rich data and one study with thin data)	Moderate confidence

	methodological limitations)	Australia, UK and USA, covering three continents)	finding is over-simplified)		
10. Veterans with a diagnosis of PTSD were just as likely to receive a family consultation regarding ACP as those without a diagnosis of PTSD<br>Study 10	Very minor methodological limitations	Serious concerns about relevance as only one study representing one country contributed to this finding	Serious concerns about adequacy (only one study offering data)	Serious concerns about adequacy (only one study offering data	Very low confidence
11. When families were involved in the care of veterans with a diagnosis of PTSD they reported unmet needs for emotional support and felt generally that their relatives were not treated with dignity and were unsatisfied with the level of care received<br>Study 22	Very minor methodological limitations	Serious concerns about relevance as only one study representing one country contributed to this finding	Serious concerns about adequacy (only one study offering data)	Serious concerns about adequacy (only one study offering data)	Very low confidence
**Advocacy**					
Having an advocate that can support a person with SMI throughout their cancer journey, including at EoL, is important and such advocates can prevent people with SMI from falling through gaps in the system[64,65,77,79]					Ungraded – non research
12. HCPs report that being referred to PC and receiving services, appears to be based on the presence or absence of a strong advocate<br>Studies 13, 15, 16	No or very minor methodological limitations (one study with no and two studies with very minor methodological limitations)	Moderate concerns about relevance (partial relevance, as the studies were from three countries only which were UK, USA and Australia)	No concerns about coherence	Minor concerns about adequacy (two studies with moderately rich data and one study with thin data)	High confidence
13. People with SMI can lack access to advocacy to help navigate the complex EoL	No or very minor methodological	Minor concerns about relevance (studies of	Moderate concerns	Minor concerns about adequacy	High confidence

trajectory due to limited social and family support and as a result they can become "lost in the system"<br>Studies 13, 15, 16, 24	limitations (one study with no and two studies with very minor methodological limitations)	HCPs representing a variety of settings and from across four countries which were Netherlands, Australia, UK and USA, covering three continents)	about coherence (data more varied and this finding is over-simplified)	(three studies with moderately rich data and one study with thin data)	
**End of life care preferences**					
ACP for people with SMI at EoL is important and mechanisms should be in place to support such people to make their own decisions, although it is recognised that this can be problematic[62,68,69,73,76,77]					Ungraded – non research
14. HCPs have concerns about negotiating EoL care preferences with people with SMI for fear that mental health symptoms may influence understanding and expectations or that such discussions may lead to further distress<br>Studies 15, 16, 21, 24	Very minor methodological limitations<br>(all studies had very minor methodological limitations)	Moderate concerns about relevance (partial relevance, as the studies were from three countries only which were Ireland, USA and Australia)	Moderate concerns about coherence (data more varied and this finding is over-simplified)	No concerns	Moderate confidence
It is important not to assume that mental capacity is lacking[15]					
15. HCPs report that they are not always comfortable with determining patients' capability to make medical decisions and that they tend to assume that mental capacity is lacking and as a result discussions aound ACP are avoided<br>Studies 13, 15, 23	No or very minor methodological limitations (two studies with no and one study with very minor methodological limitations)	Moderate concerns about relevance (partial relevance, as the studies were from three countries only which were Australia, UK and USA)	Minor concerns about coherence (data reasonably consistent within and across all studies)	Minor concerns about adequacy (two studies with moderately rich data and one study with thin data)	Moderate confidence

16. Findings from scenario-based preferences regarding medical ACP suggest that people with SMI are able to designate treatment preferences in response to EoL and are open to discussing EoLC<br>Studies 20, 29, 30	No or very minor methodological limitations (two studies with no and one study with very minor methodological limitations)	Moderate concerns about relevance (partial relevance, as the studies were from three countries only which were Belgium, USA and Canada)	Moderate concerns about coherence (data more varied and this finding is over-simplified)	No concerns	Moderate confidence
17. People with SMI conditions are capable of completing ACPs for EoL but even where legislation is in place to support this it rarely appears to be standard practice<br>Studies 12, 24, 28	Minor methodological limitations (two studies with very minor and one study with serious methodological limitations)	Moderate concerns about relevance (partial relevance, as the studies were from two countries only which were Netherlands and USA)	Moderate concerns about coherence (data more varied and this finding is over-simplified)	Minor concerns about adequacy (two studies with moderately rich data and one study with thin data)	Moderate confidence
18. A lack of confidence to have open communication and experience amongst HCPs, especially when working with homeless people, may explain the absence of ACP for those with SMI at EoL<br>Studies 12, 15,19, 21<br><br>Results across studies were mixed regarding the likelihood of people with a diagnosis of SMI having completed an advance directive (Grade – Low)	No or very minor methodological limitations (one study with no and three studies with very minor methodological limitations)	Moderate concerns about relevance (partial relevance, as the studies were from three countries only which were Australia, UK and Canada)	Moderate concerns about coherence (data more varied and this finding is over-simplified)	No concerns	Moderate confidence

The synthesis findings presented here are drawn from the wider thematic synthesis undertaken for this review. The themes identified were summarised into evidence statements, as illustrated in this table

Key: ACP: Advanced Care Planning; EoL: End of Life; EoLC: End of Life Care; HCP: Health Care Professional; MH: Mental Health; PC: Palliative Care; PTSD: Post-Traumatic Stress Disorder; SMI: Severe Mental Illness; SPC: Specialist Palliative Care.

CERQual qualitative evidence profile, synthesis 4: Living with severe mental illness

Review Finding<br><br>Studies Contributing to the Review Finding	Assessment of Methodological Limitations	Assessment of Relevance	Assessment of Coherence	Assessment of Adequacy	Overall CERQual Assessment of Confidence
**Living with severe mental illnesses: Complexities of end of life care**					
Providing EoLC to people with SMI is challenged by patients' behaviour associated with their mental health difficulties[68,77]					Ungraded – non research
1. Challenging behaviours, communication issues and side effects of combining SMI and EOL medications make it difficult to address MH issues at EoL<br>Studies 13-17, 24	Very minor methodological limitations (all studies had very minor methodological limitations)	Minor concerns about relevance (studies of HCPs and patients representing a variety of settings and from across four countries which were Netherlands Australia, UK and USA, covering three continents)	Moderate concerns about coherence (data more varied and this finding is over-simplified)	Minor concerns about adequacy (six studies with rich data and one study with thin data)	Moderate confidence
**Living with severe mental illnesses: Familiarity and trust**<br>**Trust and rapport**					
2. Early referral to PC helps build trust and rapport between staff and people with SMI at the EoL<br>Studies 13-15, 17, 19, 25, 28	No or very minor methodological limitations (four studies with very minor and three studies with no methodological limitations)	Minor concerns about relevance (studies of HCPs and patients representing a variety of settings and from across five countries which were Canada, Australia, Ireland, UK and USA, covering three continents)	Moderate concerns about coherence (data more varied and this finding is over-simplified)	Minor concerns about adequacy (six studies with rich data and two studies with thin data)	Moderate confidence
**Supporting people in familiar environments**					

3. Although people with SMI often leave environments with which they are familiar at the EoL, MH and PC staff can work together to support people without the need for moving<br>Studies 14, 19, 21, 25	No or very minor methodological limitations (three studies with very minor and one study with no methodological limitations)	Minor concerns about relevance (studies of HCPs and patients representing a variety of settings and from across four countries which were Canada, Australia, Ireland and UK covering three continents)	Moderate concerns about coherence (data more varied and this finding is over-simplified)	No concerns	Moderate confidence
Deteriorations in EoL-related physical health commonly results in the homeless person being transferred to hospital in crisis where their needs are poorly met[77]		Moderate concerns about relevance (partial relevance, as the studies were from two countries only			Ungraded – non research
4. Hostel staff report that they try to ensure that PC can be provided in a familiar environment for as long as possible as they feel the benefits outweigh the challenges<br>Studies 19, 21	No or very minor methodological limitations Moderate confidence (one study with very minor and one study with no methodological limitations)	Moderate concerns about relevance (partial relevance, as the studies were from two countries only which were UK and Canada	Minor concerns about coherence (data reasonably consistent within and across all studies)	Very minor concerns about adequacy (two studies with rich data)	Moderate confidence
**Living with severe mental illnesses: Recognising physical decline**<br>**Identifying signs of declining health**					
The physical health needs and signs of physical deterioration of those with pre-existing SMI may not be identified and as a result care is often inadequate[64,77,79]					Ungraded – non research
5. Staff report that people with SMI are not always able to recognise their own declining	No or very minor methodological	Minor concerns about relevance (studies of	Moderate concerns	Minor concerns about adequacy	Moderate confidence

health and because of previous unsatisfactory healthcare encounters it is felt that they often present late to services<br>Studies 13, 15, 24, 25	limitations (Three studies with very minor and one study with no methodological limitations)	HCPs and patients representing a variety of settings and from across four countries which were Australia, Ireland, Netherlands and UK covering three continents)	about coherence (data more varied and this finding is over-simplified)	(three studies with rich data and one study with thin data)	
**The impact of late diagnosis**					
6. The timely provision of PC can be hampered when people with SMI (especially those who are homeless), are not identified as approaching EOL until late diseases of life-limiting physical disease<br>Studies 15, 21, 24	Very minor methodological limitations (all studies had very minor methodological limitations)	Moderate concerns about relevance (partial relevance, as the studies were from three countries only which were Netherlands, Australia and USA)	No concerns about coherence	No concerns about adequacy	High confidence
**Identifying an EoLC trajectory for those who are homeless**					
7. People who are homeless are often more concerned with day-to-day survival than with keeping appointments with HCPs and this makes it difficult to identify EoL trajectories and to provide PC<br>Studies 19, 21	No or very minor methodological limitations (one study with very minor and one study with no methodological limitations)	Moderate concerns about relevance (partial relevance, as the studies were from two countries only which were UK and Canada	Moderate concerns about coherence (data more varied and this finding is over-simplified)	Very minor concerns about adequacy (two studies with rich data)	Moderate confidence

The synthesis findings presented here are drawn from the wider thematic synthesis undertaken for this review. The themes identified were summarised into evidence statements, as illustrated in this table

Key: EoL: End of Life; EoLC: End of life Care: HCP: Health Care Professional; MH: Mental Health; PC: Palliative Care; SMI: Severe Mental Illness.
