# Supplementary material for: End of life care for people with severe mental illness: Mixed methods systematic review and thematic synthesis (the MENLOC study)
Source: Palliat Med. 2021 Sep 3;35(10):1747–60. doi: 10.1177/02692163211037480 (PMC8637363; doi:10.1177/02692163211037480)
Supplement: sj-pdf-5-pmj-10.1177_02692163211037480 – Supplemental material for End of life care for people with severe mental illness: Mixed methods systematic review and thematic synthesis (the MENLOC study) [file sj-pdf-5-pmj-10.1177_02692163211037480.pdf]

Summary of findings and GRADE ratings from retrospective cohort studies

Theme and sub theme	Effect	Effect Size	Study	GRADE
Structure of the system: No right place to die				
Dying at home	No differences in the likelihood of home deaths between decedents with and without previous mental illness	OR 1.02 95% CI 0.92-1.14	Lavin et al. 2017[1]	Very Low
Dying at home	Decedents with schizophrenia diagnoses were 65% less likely to have died in a nursing home compared to those without	OR 0.35, 95% CI 0.28-0.44	Martens et al. 2013[2]	Very Low
Dying in a nursing home or residential facility	When all causes of death were considered, having any type of pre-existing mental illness was associated with 27% higher odds of dying in a nursing home compared to those without	OR 1.27, 95% CI 1.09-1.49	Lavin et al. 2017[1]	Very Low
Dying in a nursing home or residential facility	In the case of cancer-specific deaths, having a schizophrenia diagnosis was associated with significantly higher odds of dying in a nursing home compared to those without	OR 49.33 95% CI 43.89–55.45	Martens et al. 2013[2]	Very Low
Dying in a nursing home or residential facility	For lung cancer deaths, having a schizophrenia diagnosis was associated with higher odds of dying in an assisted living facility compared to those without	OR 3.16, 95% 1.33-7.49	Ganzini et al. 2010[3]	Very Low
Dying in a nursing home or residential facility	For lung cancer deaths, having a schizophrenia diagnosis was associated with higher odds of dying in a skilled nursing unit compared to those without	OR 1.31, 95% CI 0.59-2.89	Ganzini et al. 2010[3]	Very Low
Dying in a hospice	No significant differences in the likelihood of dying in a hospice between those with and without a history of previous mental illness	OR 0.98 95% CI 0.79-1.22	Lavin et al. 2017[1]	Very Low
Contexts of care: Health care services and treatment utilisation in the last year of life				
Ambulatory visits to GP or medical specialists	Rates of ambulatory visits to the GP and medical specialists, and whether this was higher or lower for those with and without schizophrenia diagnoses who had died from cancer. No differences were observed in rates of ambulatory visits to the GP within six months of death	aRR 1.03, 95% CI 0.96-1.11	Chochinov et al. 2012[4]	Very Low
Ambulatory visits to GP or medical specialists	in the six months to death decedents with schizophrenia diagnoses were 39% less likely to visit medical specialists compared to those without	aRR 0.61, 95% CI 0.54-0.69	Chochinov et al. 2012[4]	Very Low
Palliative care services	Decedents with SMI were 71% less likely to access SPC services (community and inpatient services) compared to those without SMI in the LYoL	RR 0.29, 95% CI 0.24-0.34	Butler and O'Brien 2018[5]	Very Low
Palliative care services	No significant differences in the overall utilisation of PC services (hospice ward card, PC consultation and hospice home care)	OR 1.03, 95% CI 0.090-1.18	Huang et al. 2018[6]	Very Low

	between those with schizophrenia diagnosis compared to those without at three months before death			
	No significant differences in the overall utilisation of PC services (hospice ward card, PC consultation and hospice home care) between those with schizophrenia diagnosis compared to those without at one month before death	OR 1.03, 95% CI 0.99-1.33	Huang et al. 2018[6]	Very Low
Palliative care services	People with SMI were 31% less likely to receive a PC consultation compared to those without during the last month of life	OR 0.59 96%CI 0.43-0.82	Huang et al. 2018[6]	Very Low
Palliative care services	Decedents with schizophrenia diagnoses and cancer were 61% more likely to have frequent admissions to PC units in the last 31 days before death	OR 1·61 95% CI 1·45–1·80	Fond et al. 2019[7]	Very Low
Palliative care services	Decedents with schizophrenia diagnoses and cancer were 152% more likely in the last three days before death than matched controls	OR 2·52, 95% CI 2·25–2·82	Fond et al. 2019[7]	Very Low
Palliative care services	Decedents with schizophrenia diagnoses and cancer were to have had longer PC follow-up before death than matched controls	ß 0·36; SD 0·03	Fond et al. 2019[7]	Very Low
Palliative care services	No significant differences in rates of hospice ward care between decedents with schizophrenia diagnoses compared to those without within one month of death	RR 1.15, 95% CI 0.99-1.33	Huang et al. 2018[6]	Very Low
Palliative care services	No significant differences in rates of hospice enrolment between decedents with schizophrenia diagnoses compared to those without within one month of death	RR 11.3, 95% CI 0.63-2.01	Ganzini et al. 2010[3]	Very Low
Palliative care services	Having depression ahead of a cancer diagnosis was found to be associated with a 19% higher hazard of hospice enrolment than those without within the 30 days of death	HR 1.19, 95% CI 1.11-1.28	McDermott et al. 2018 [8]	Very Low
Palliative care services	Decedents with schizophrenia diagnoses had significantly longer hospice stays than those without	MD 44, 95% CI 5.16-82.84	Ganzini et al. 2010[3]	Very Low
Palliative care services	Having depression ahead of a cancer diagnosis was associated with a 29% increased likelihood of longer hospice stays of 90 or more days compared to those without	aOR, 1.29, 95% CI 1.06-1.58	McDermott et al. 2018 [8]	Very Low
Palliative care services	Decedents with schizophrenia diagnoses (where the cause of death meant that they were amenable to PC) were 66% less likely to receive hospital-based SPC	RR 0.34, 95% CI 0.26-0.44	Spilsbury et al. 2018[9]	Very Low
Palliative care services	Decedents with schizophrenia diagnoses (where the cause of death meant that they were amenable to PC) were 62% less likely to have received SPC in the community compared to those without in the LYoL	OR 0.38, 95% CI 0.30-0.48	Spilsbury et al. 2018[9]	Very Low

Palliative care services	being enrolled in community-based SPC was associated with a 50% increased rate of hospital admission	HR 1.5, 95% CI 1.1-2.1	Spilsbury et al. 2018[9]	Very Low
Palliative care services	There was no change in the rate of ED presentation as a result of being enrolled in community-based SPC	HR 1.2 95% CI 0.8-1.7	Spilsbury et al. 2018[9]	Very Low
Palliative care services	Decedents with schizophrenia diagnoses who died of cancer were 29% less likely to have used SPC in the community than those without within six months of death	aRR 0.71, 95% CI 0.62-0.81	Chochinov et al. 2012[4]	Very Low
Palliative care services	Decedents with schizophrenia diagnoses who died of cancer received significantly fewer days of SPC in the community on average	59.3 days / 76.2 days, p<0.05	Chochinov et al. 2012[4]	Very Low
Palliative care services	There were no significant differences in rates of admission to SPC in the month before death between decedents with schizophrenia diagnoses where the cause of death was cancer compared to those without	OR 1.11, 95% CI 0.89-1.39	Huang et al. 2018[6]	Very Low
Palliative care services	There were no significant differences in rates of admission to SPC in the three months before death between decedents with schizophrenia diagnoses where the cause of death was cancer compared to those without	OR 1.21, 95% CI 0.99-1.48	Huang et al. 2018[6]	Very Low
Long term institutional care	Decedents with schizophrenia diagnoses who died of cancer had a significantly increased rate of use of long-term institutional care compared with those without within six months of death as demonstrated by a greater percentage residing in a nursing home	aRR: 4.28 95% CI 3.55-5.16	Chochinov et al. 2012[4]	Very Low
Long term institutional care	Decedents with schizophrenia diagnoses who died of cancer had significantly longer lengths of stay compared to those without schizophrenia diagnoses in the six months before death as demonstrated by the rate of days residing in a nursing home (per person)	aRR 5.19 95% CI 3.60-7.78	Chochinov et al. 2012[4]	Very Low
Long term institutional care	Decedents with schizophrenia diagnoses who died in a nursing home had significantly lower rates of inpatient hospital days compared to those without within with six months of death	aRR 0.80, 95% CI 0.67-0.95	Martens et al. 2013[2]	Very Low
Long term institutional care	No significant differences were shown between cohorts for rates of GP visits	aRR 1.14, 95% CI 0.89-1.20	Martens et al. 2013[2]	Very Low
Long term institutional care	No significant differences were shown between cohorts for rates of specialist visits	aRR 1.14, 95% CI 0.97-1.33	Martens et al. 2013[2]	Very Low
Long term institutional care	No significant differences were shown between cohorts for hospital separation rates	RR 0.93, 95% CI 0.82-1.06	Martens et al. 2013[2]	Very Low

Acute care	Decedents with schizophrenia diagnoses who died of cancer had a significant reduction in the rate of inpatient hospital separations compared to those without within six months prior to death	aRR 0.79, 95% CI 0.73-0.86	Chochinov et al. 2012[4]	Very Low
Acute care	Across all causes of death, decedents with pre-existing psychiatric illnesses were 41% less likely to have a hospital admission compared to those without within one month of death	OR 0.59, 95% CI 0.51-0.68	Lavin et al. 2017[1]	Very Low
Acute care	Decedents with schizophrenia diagnoses were 27% less likely to have had more than one admission to acute care during the last 31 days of life	aOR 0.73, 95% CI 0.67-0.80	Fond et al. 2019[7]	Very Low
Acute care	decedents with schizophrenia diagnoses had a 21% reduction in the hospital admission rate within six months of death compared to those without	aRR 0.85, 95% CI 0.77-0.94	Chochinov et al. 2012[4]	Very Low
Acute care	Decedents with a schizophrenia diagnosis had a 40% reduction in the rate of admission compared to those without (except for breast cancer) within the LYoL	aHR 0.6, 95% CI 0.5-0.8	Spilsbury et al. 2018[9]	Very Low
Acute care	Lower rates of hospital admission were also observed for decedents with schizophrenia diagnoses who had heart failure	aHR 0.6 95% CI 0.5-0.8	Spilsbury et al. 2018[9]	Very Low
Acute care	Lower rates of hospital admission were also observed for decedents with schizophrenia diagnoses who had cirrhosis/liver disease	aHR 0.5, 95% CI 0.3-1.2	Spilsbury et al. 2018[9]	Very Low
Acute care	Lower rates of hospital admission were also observed for decedents with schizophrenia diagnoses who had renal disease/dialysis	aHR: 0.6, 95% CI 0.3-1.2	Spilsbury et al. 2018[9]	Very Low
Acute care	No differences in rates of hospital admission were found for those for decedents with schizophrenia diagnoses who had died from chronic lower respiratory disease	HR 0.7, 95% CI 0.4-1.2	Spilsbury et al. 2018[9]	Very Low
Acute care	No difference in length of stay once admitted for those with schizophrenia diagnoses who died of cancer and those without in the six months to death	(aRR 1.00, 95% CI 0.85-1.19)	Chochinov et al. 2012[4]	Very Low
Acute care	No difference in length of stay once admitted for those with pre-existing mental illness and those without within one month of death across all causes of death	(RR 0.93, 95% CI 0.86-1.00)	Lavin et al. 2017[1]	Very Low
Acute care	The median length of stay in acute care during the last 31 days of life was higher for decedents with schizophrenia diagnoses who died of cancer	days (median (IQR) 16 (8-28) vs 22 (11-31) p<0.001)	Fond et al. 2019[7]	Very Low
Intensive care unit admissions	When all causes of death were considered decedents with pre-existing psychiatric illnesses were 59% less likely to be have been	OR 0.41, 95% CI 0.35-0.48	Lavin et al. 2017[1]	Very Low

	admitted for care in an ICU compared to those without within one month of death			
Intensive care unit admissions	Decedents with pre-cancer depression were 22% less likely to be admitted for care in an ICU compared to those without within one month of death when the cause of death was lung cancer	aOR 0.78, 95% CI 0.67-0.90	McDermott et al. 2018 [8]	Very Low
Intensive care unit admissions	Having schizophrenia diagnoses and having died of cancer was associated with a 21% increased likelihood of having been admitted for care in an ICU admission compared to those without within one month of death	OR 1.21, 95% CI 1.07-1.36	Huang et al. 2018[6]	Very Low
Intensive care unit admissions	No association between having schizophrenia diagnoses and having died of cancer and at least one ICU admission during the last 31 days of life	aOR 0.92; 95% CI 0.91-1.09	Fond et al. 2019[7]	Very Low
Intensive care unit admissions	Decedents with pre-existing mental illnesses spent significantly fewer days in the ICU compared to those without pre-existing mental illness in the one month prior to death	RR 0.88, 95% CI 0.79-0.97	Lavin et al. 2017[1]	Very Low
Emergency department visits	Across all causes of death, having pre-existing psychiatric illnesses was associated with a 64% increased likelihood of visiting the ED at least once compared to those without within one month of death	OR 1.64 95% CI 1.30-2.08	Lavin et al. 2017[1]	Very Low
Emergency department visits	Having pre-cancer depression was associated with a 22% reduction in the likelihood of visiting the ED at least once compared to those without pre-cancer depression within one month of death	aOR 0.78, 95% CI 0.62-0.98	McDermott et al. 2018 [8]	Very Low
Emergency department visits	No associations between having a schizophrenia diagnosis and attending the ED at least once during the last 31 days before death	OR 0.93, 95% CI 0.91-1.09	Fond et al. 2019[7]	Very Low
Emergency department visits	Decedents with schizophrenia diagnoses compared to those without, there have been found to be significantly higher rates of attending the ED within one month of death for those dying from ischaemic heart disease	HR 1.1 95% CI 1.0-1.4	Spilsbury et al. 2018[9]	Very Low
Emergency department visits	Decedents with schizophrenia diagnoses compared to those without, there have been found to be significantly higher rates of attending the ED within one month of death for those dying from heart failure	HR 1.0, 95% CI 0.8-1.3	Spilsbury et al. 2018[9]	Very Low
Emergency department visits	Decedents with schizophrenia diagnoses compared to those without, there have been found to be significantly higher rates of attending the ED within one month of death for those dying from chronic lower respiratory disease	HR 1.1 96% CI 0.8-1.3)	Spilsbury et al. 2018[9]	Very Low

Emergency department visits	Decedents with schizophrenia diagnoses compared to those without, there have been found to be significantly higher rates of attending the ED within one month of death for those dying from renal disease/dialysis	HR 1.1 95% CI 0.8-1.5[9]	Spilsbury et al. 2018[9]	Very Low
Emergency department visits	Decedents with schizophrenia diagnoses compared to those without, there have been found to be significantly higher rates of attending the ED within one month of death for those dying from cirrhosis/liver disease	HR 1.0, 95% CI 0.8-1.2	Spilsbury et al. 2018[9]	Very Low
Emergency department visits	Decedents with schizophrenia diagnoses compared to those without, there have been found to be significantly higher rates of attending the ED within one month of death for those dying from cancer	HR 1.2, 95% CI 1.1-1.4	Spilsbury et al. 2018[9]	Very Low
Invasive interventions	no significant difference in orders about CPR existed between decedents with schizophrenia diagnoses and those without within six months of death at a VA medical centre, when the cause of death was cancer	aRR 1.03, 95% CI 0.96-1.11	Ganzini et al. 2010[3]	Very Low
Invasive interventions	decedents with schizophrenia diagnoses where the cause of death was cancer were significantly more likely to have had CPR than those without	OR 1.34, 96% CI 1.15-1.57	Huang et al. 2018[6]	Very Low
Invasive interventions	decedents with schizophrenia diagnoses where the cause of death was cancer were significantly more likely to have had endotracheal intubation than those without	OR 1.22, 95% CI 1.08–1.38	Huang et al. 2018[6]	Very Low
Invasive interventions	Decedents with schizophrenia diagnoses where the cause of death was cancer were significantly more likely to have had mechanical ventilation than those without	OR 1.15, 95% CI 1.03–1.29),	Huang et al. 2018[6]	Very Low
Invasive interventions	Decedents with schizophrenia diagnoses where the cause of death was cancer were significantly more likely to have had urinary catheterization than those without	(OR 1.19, 95% CI 1.07–1.32	Huang et al. 2018[6]	Very Low
Invasive interventions	Decedents with schizophrenia diagnoses where the cause of death was cancer were significantly more likely to have had feeding tubes than those without	OR 1.41, 95% CI 1.26–1.58	Huang et al. 2018[6]	Very Low
Invasive interventions	No significant differences were found in artificial nutrition, during the last 31 days before death amongst those with schizophrenia diagnoses compared to those without	aOR 0.85, 95% CI 0.69-1.05	Fond et al. 2019[7]	Very Low
Invasive interventions	No significant differences were found in tracheal intubation during the last 31 days before death amongst those with schizophrenia diagnoses compared to those without	aOR 0.84, 95% CI 0.69-1.13	Fond et al. 2019[7]	Very Low

Invasive interventions	No significant differences were found in mechanical ventilation during the last 31 days before death amongst those with schizophrenia diagnoses compared to those without	aOR 1.00, 95% CI 0.88-1.14	Fond et al. 2019[7]	Very Low
Invasive interventions	No significant differences were found in gastrostomy during the last 31 days before death amongst those with schizophrenia diagnoses compared to those without	aOR 0.67, 95% CI 0.34-1.32	Fond et al. 2019[7]	Very Low
Invasive interventions	No significant differences were found in dialysis during the last 31 days before death amongst those with schizophrenia diagnoses compared to those without	aOR 0.80, 95% CI 0.19-1.18	Fond et al. 2019[7]	Very Low
Invasive interventions	No significant differences were found in CPR during the last 31 days before death amongst those with schizophrenia diagnoses compared to those without	aOR 0.47, 95% CI 0.19-1.18	Fond et al. 2019[7]	Very Low
Invasive interventions	No significant differences were found in having at least one air extraction (sterile) chamber admission during the last 31 days before death amongst those with schizophrenia diagnoses compared to those without	aOR 0.48, 95% CI 0.23-0.99	Fond et al. 2019[7]	Very Low
Invasive interventions	Decedents with schizophrenia diagnoses and cancer were less likely to have undergone surgery in the last 31 days before death	OR 0·71, 95% CI 0·63–0·80	Fond et al. 2019[7]	Very Low
Invasive interventions	Decedents with schizophrenia diagnoses and cancer were less likely to have undergone blood transfusions in the last 31 days before death	OR 0·72, 95% CI 0·64–0·83	Fond et al. 2019[7]	Very Low
Invasive interventions	Veterans with pre-existing, but unspecified, MH conditions where over half had a terminal condition of cancer (30.1%) or heart disease (20.1%) at the time of death were 20% less likely to have an IV infusion than those without	OR0.80, p=0.001	Huang et al. 2017[10]	Very Low
Invasive interventions	Veterans with pre-existing, but unspecified, MH conditions where over half had a terminal condition of cancer (30.1%) or heart disease (20.1%) at the time of death were 23% more likely to have a do not resuscitate order than those without	OR 1.23, p=0.002	Huang et al. 2017[10]	Very Low
Invasive interventions	Veterans with pre-existing, but unspecified, MH conditions where over half had a terminal condition of cancer (30.1%) or heart disease (20.1%) at the time of death were 23% more likely to have physical restraints) than those without	OR 1.23, p=0.004	Huang et al. 2017[10]	Very Low
Invasive interventions	Decedents with schizophrenia diagnoses who died of cancer were 267% more likely to have POLST in place	OR 3.67, 95% CI 1.38-9.72	Ganzini et al. 2020[3]	Very Low
Chemotherapy	Decedents with schizophrenia diagnoses when the cause of death was cancer were 45% less likely to have chemotherapy compared to those without within one month of death	OR 0.55, 95% CI 0.48-0.63	Huang et al. 2017[10]	Very Low

Chemotherapy	Decedents with schizophrenia diagnoses when the cause of death was cancer were 47% less likely in the last 14 days of life	aOR 0·53, 95% CI: 0·46–0·62	Fond et al. 2019[7]	Very Low
Chemotherapy	No association was found between having depression ahead of a lung cancer diagnosis and not having depression ahead of diagnosis in the use of chemotherapy in the last 14 days of life	aOR 0.89, 95% CI 0.74-1.07	McDermott et al. 2018 [8]	Very Low
Advanced diagnostic examinations	Decedents with schizophrenia diagnoses where the cause of death was cancer were less likely to have had computed tomography, magnetic resonance imaging/sonography compared to those without within one month of death	OR 0.80, 95% CI 0.71-0.89	Huang et al. 2018[6]	Very Low
Advanced diagnostic examinations	Decedents with schizophrenia diagnoses where the cause of death was cancer were less likely to have had bone scans compared to those without within one month of death	OR 0.62, 95% CI 0.50-0.76	Huang et al. 2018[6]	Very Low
Advanced diagnostic examinations	Decedents with schizophrenia diagnoses where the cause of death was cancer were less likely to have had positron emission tomography scans compared to those without within one month of death[6]	OR 0.37, 95% CI 0.15-0.96	Huang et al. 2018[6]	Very Low
Advanced diagnostic examinations	There were no differences in the use of pan-endoscopy for decedents with and without schizophrenia diagnoses where the cause of death was cancer	OR 0.98, 95% CI 0.84-1.15	Huang et al. 2018[6]	Very Low
Advanced diagnostic examinations	There were no differences in the use of or colonoscopy for decedents with and without schizophrenia diagnoses where the cause of death was cancer	OR 1.02, 95% CI 0.74-1.41	Huang et al. 2018[6]	Very Low
Advanced diagnostic examinations	Decedents with schizophrenia diagnoses and cancer were less likely to have had imaging) in the last 31 days before death compared to those without	OR 0·66, 95% CI 0·59–0·73	Fond et al. 2019[7]	Very Low
Advanced diagnostic examinations	Decedents with schizophrenia diagnoses and cancer were less likely to have had endoscopy in the last 31 days before death compared to those without	OR 0·85, 95% CI 0·74–0·97	Fond et al. 2019[7]	Very Low
Use of medications at the end of life	Decedents with schizophrenia diagnoses who died of cancer were 44% less likely to have used analgesia than those without in the six months prior to death, as demonstrated by the total days and rate per person-year of receiving analgesics	aRR 0.66, 95% CI 0.54-0.81	Chochinov et al. 2012[4]	Very Low
Use of medications at the end of life	there was no significant difference in the number of different drugs used between those with schizophrenia diagnoses who died from cancer and those without within six months of death as demonstrated by the number of different drugs (per person-year, for those on at least one prescription in the 6 months)	aOR1.02 9 95% CI 0.96-1.08	Chochinov et al. 2012[4]	Very Low

Use of medications at the end of life	Veterans with schizophrenia diagnoses where the cause of death was cancer compared to those without were no less likely to receive opioid medications before hospice enrolment in the last six months of life (	OR 0.64, 95% CI 0.33-1.26	Ganzini et al. 2020[3]	Very Low
Use of medications at the end of life	There was a significant association between a lifetime diagnosis of PTSD and the medications needed at the EoL, where there was found to be a higher utilization of medications at the EoL among terminally ill veterans with PTSD (in particular hypnotics and antidepressants) compared to those without PTSD	72% versus 40%, p=0.0005	Kelley-cook et al. 2016[11].	Very Low
**Contexts of care: Meeting individual and family needs**				Very Low
End of life care preferences	No differences in the likelihood of veterans with schizophrenia diagnoses and advanced cancer completed an AD compared with those without schizophrenia diagnoses	OR 1.29, 95% CI 0.72-2.3)	Ganzini et al. 2020[3]	Very Low
End of life care preferences	aving a diagnosis of severe mental illness was significantly associated with 24% lower odds of having any AD in a nursing home after controlling for a variety of resident and family characteristics	aOR 76.95, 95% CI 0.66-0.87	Cai et al. 2011[12]	Very Low

Key: 95% CI: 95% confidence intervals; AD: advance directive(s); AoR: adjusted odds ratio; aRR: adjusted relative risk; ED: emergency department; GP: general practitioner; GRADE: Grading of Recommendations, Assessment, Development and Evaluation HR: hazard ratio; ICU: Intensive care unit; LYoL: IQR: interquartile range; last year of life; MD: mean difference; MH: mental heath; OR: odds ratio; POLST: Physician Orders for Life Sustaining Treatment; PTSD: post-traumatic stress disorder; RR: relative risk; SD: standard deviation; SMI: severe mental illness; SPC: specialist palliative care
